# A fast and agnostic method for bacterial genome-wide association studies: Bridging the gap between k-mers and genetic events

**DOI:** 10.1371/journal.pgen.1007758

**Published:** 2018-11-12

**Authors:** Magali Jaillard, Leandro Lima, Maud Tournoud, Pierre Mahé, Alex van Belkum, Vincent Lacroix, Laurent Jacob

**Affiliations:** 1 bioMérieux, Marcy l’Étoile, France; 2 Univ Lyon, Université Lyon 1, CNRS, Laboratoire de Biométrie et Biologie Evolutive UMR5558 F-69622 Villeurbanne, France; 3 EPI ERABLE - Inria Grenoble, Rhône-Alpes, France; Imperial College London, UNITED KINGDOM

## Abstract

Genome-wide association study (GWAS) methods applied to bacterial genomes have shown promising results for genetic marker discovery or detailed assessment of marker effect. Recently, alignment-free methods based on k-mer composition have proven their ability to explore the accessory genome. However, they lead to redundant descriptions and results which are sometimes hard to interpret. Here we introduce DBGWAS, an extended k-mer-based GWAS method producing interpretable genetic variants associated with distinct phenotypes. Relying on compacted De Bruijn graphs (cDBG), our method gathers cDBG nodes, identified by the association model, into subgraphs defined from their neighbourhood in the initial cDBG. DBGWAS is alignment-free and only requires a set of contigs and phenotypes. In particular, it does not require prior annotation or reference genomes. It produces subgraphs representing phenotype-associated genetic variants such as local polymorphisms and mobile genetic elements (MGE). It offers a graphical framework which helps interpret GWAS results. Importantly it is also computationally efficient—experiments took one hour and a half on average. We validated our method using antibiotic resistance phenotypes for three bacterial species. DBGWAS recovered known resistance determinants such as mutations in core genes in *Mycobacterium tuberculosis*, and genes acquired by horizontal transfer in *Staphylococcus aureus* and *Pseudomonas aeruginosa*—along with their MGE context. It also enabled us to formulate new hypotheses involving genetic variants not yet described in the antibiotic resistance literature. An open-source tool implementing DBGWAS is available at https://gitlab.com/leoisl/dbgwas.

## Author summary

Genome-wide association studies (GWAS) help explore the genetic bases of phenotype variation in a population. Our objective is to make GWAS amenable to bacterial genomes. These genomes can be too different to be aligned against a reference, even within a single species, making the description of their genetic variation challenging. We test the association between the phenotype and the presence in the genomes of DNA subsequences of length *k* – the so-called k-mers. These k-mers provide a versatile descriptor, allowing to capture genetic variants ranging from local polymorphisms to insertions of large mobile genetic elements. Unfortunately, they are also redundant and difficult to interpret. We rely on the compacted De Bruijn graph (cDBG), which represents the overlaps between k-mers. A single cDBG is built across all genomes, automatically removing the redundancy among consecutive k-mers, and allowing for a visualisation of the genomic context of the significant ones. We provide a computationally efficient and user-friendly implementation, enabling non-bioinformaticians to carry out GWAS on thousands of isolates in a few hours. This approach was effective in catching the dynamics of mobile genetic elements in *Staphylococcus aureus* and *Pseudomonas aeruginosa* genomes, and retrieved known local polymorphisms in *Mycobacterium tuberculosis* genomes.

## Introduction

The aim of Genome-Wide Association Studies (GWAS) is to identify associations between genetic variants and a phenotype observed in a population. They have recently emerged as an important tool in the study of bacteria, given the availability of large panels of bacterial genomes combined with phenotypic data [1–7].

GWAS rely on a representation of the genomic variation as numerical factors. The most common approaches are based on single nucleotide polymorphisms (SNPs), defined by aligning all genomes of the studied panel against a reference genome [1, 3, 4] or against a pangenome built from all the genes identified by annotating the genomes [8], and on gene presence/absence, using a pre-defined collection of genes [5, 7]. The use of a reference genome becomes unsuitable when working on bacterial species with a large accessory genome—the part of the genome which is not present in all strains. On the other hand, methods focusing on genes are unable to cover variants in noncoding regions, including those related to transcriptional and translational regulation [9, 10]. Moreover, some poorly studied species still lack a representative annotation [11].

To circumvent these issues and make bacterial genomes amenable to GWAS, recent studies have relied on k-mers: all nucleotide substrings of length *k* found in the genomes [2, 5, 6]. The presence of k-mers in genomes can account for diverse genetic events such as the acquisition of SNPs, (long) insertions/deletions and recombinations. Unlike SNP- or gene-based approaches, k-mer analyses do not require a reference genome or any assumption on the nature of the causal variants and can even be performed without assembling the genome sequences [12].

While k-mers can reflect any genomic variation in a panel, they do not themselves represent biological entities. Translating the result of a k-mer-based GWAS into meaningful genetic variants typically requires mapping a large and redundant set of short sequences [2, 5, 6, 13]. Recent studies have suggested reassembling the significantly associated k-mers to reduce redundancy and retrieve longer marker sequences [6, 13]. Nonetheless, k-mer representation often loses in interpretability what it gains in flexibility, and the best way to encode the genomic variation in bacterial GWAS is not yet clearly defined [14, 15].

**Fig 1:**
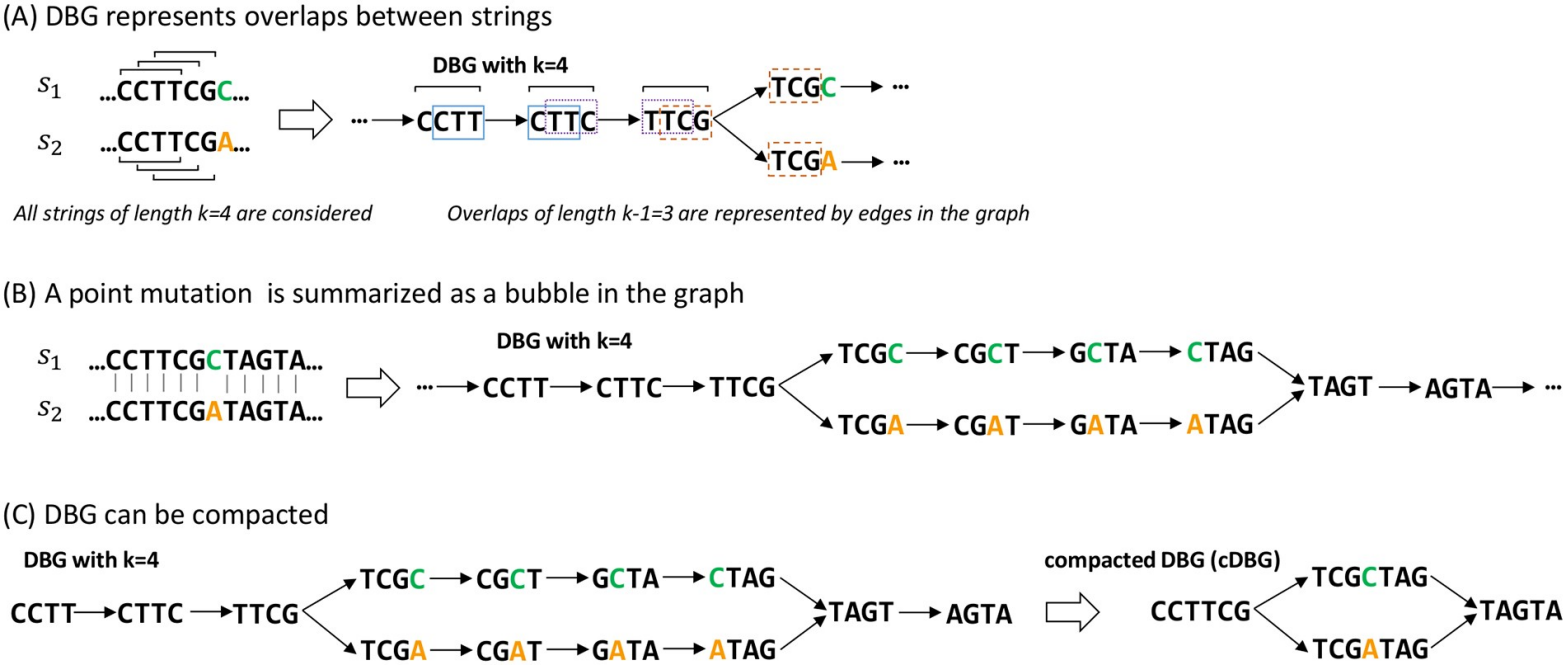
Compacted DBG construction over a set of sequences differing by a single point mutation. In this example two sequences *s*_1_ and *s*_2_ of length 12 differ by a single letter. (A) All k-mers (*k* = 4) present in these sequences are listed. A link is drawn between two k-mers when the *k* − 1 = 3 last nucleotides of the first k-mer equal the 3 first nucleotides of the second k-mer. (B) The bubble pattern represents the SNP C to A; each branch of the bubble represents an allele. (C) Linear paths of the graph are compacted; the compacted DBG of the example only contains four nodes (unitigs) and represents the same variation as the original DBG, which contained 13 nodes (k-mers).

Our approach, coined DBGWAS, for *De Bruijn Graph GWAS*, bridges the gap between, on the one hand, SNP- and gene-based representations lacking the right level of flexibility to cover complete genomic variation, and, on the other hand, k-mer-based representations which are flexible but not readily interpretable. We rely on De Bruijn graphs [16] (DBGs), which are widely used for *de novo* genome assembly [17, 18] and variant calling [12, 19]. These graphs connect overlapping k-mers (here DNA fragments), yielding a compact summary of all variations across a set of genomes. Fig 1 illustrates the construction of such a graph for a simple example, where the only variation among the aligned genomes is a point mutation. DBGs also accommodate more complex disparities including rearrangements and insertions/deletions (S1 Fig).

DBGWAS relies on the ability of compacted DBGs (cDBGs) to eliminate local redundancy, reflect genomic variations, and characterise the genomic environment of a k-mer at the population level. More precisely, we build a single cDBG from all the genomes included in the association study (in practice, up to thousands). The graph nodes—called unitigs—represent, by construction, sequences of variable length and are at the right level of resolution for the set of genomes considered, taking into account adaptively the genomic variation. The unitigs are individually tested for association with the phenotype, while controlling for population structure. The unitigs found to be phenotype-associated are then localised in the cDBG. Subgraphs induced by their genomic environment are extracted. They often provide a direct interpretation in terms of genetic events which results from the integration of three types of information: 1) the *topology* of the subgraph, reflecting the nature of the genetic variant, 2) the *metadata* represented by node size and colour, allowing us to identify which unitigs in the subgraph are associated to a particular phenotype status, and 3) an optional sequence *annotation* helping to detect unitig mapping to—or near—a known gene.

We benchmarked our novel method using several antibiotic resistance phenotypes within three bacterial species of various degrees of genome plasticity: *Mycobacterium tuberculosis*, *Staphylococcus aureus* and *Pseudomonas aeruginosa*. The subgraphs built from significant unitigs described SNPs or insertions/deletions in both core and accessory regions, and were consistent with results obtained with a resistome-based association study. In addition, novel genotype-to-phenotype associations were also suggested.

## Results

**Fig 2:**
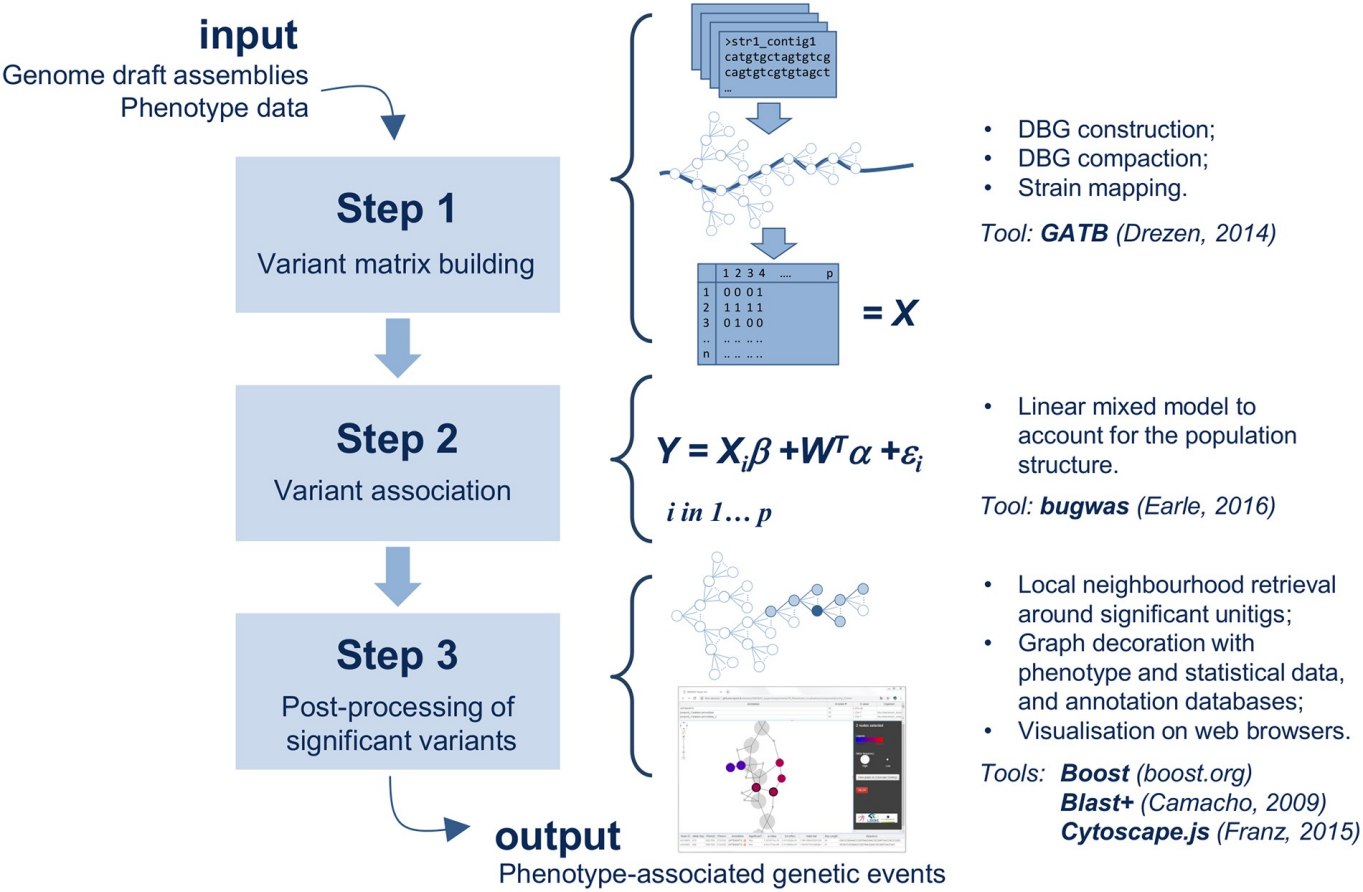
DBGWAS pipeline. DBGWAS takes as input draft assemblies and phenotype data for a panel of bacterial strains. A variant matrix *X* is built in *step* 1 using cDBG nodes (called unitigs). Variants are tested in *step* 2 using a linear mixed model taking into account the population structure. Significant variants are post-processed in *step* 3 to provide an interactive interface assisting their interpretation.

We developed DBGWAS, available at https://gitlab.com/leoisl/dbgwas, and validated it on panels for several bacterial species for which genome sequences and antibiotic resistance phenotypes were available. DBGWAS comprises three main steps: it first builds a variant matrix, where each variant is a pattern of presence/absence of unitigs in each genome. Each variant is then tested for association with the phenotype using a linear mixed model, adjusting for the population structure. Finally, it uses the cDBG neighbourhood of significantly associated unitigs as a proxy for their genomic environment. DBGWAS outputs a set of such subgraphs ordered by min_*q*_, which is the smallest q-value observed over unitigs in each subgraph. The top subgraphs therefore represent the genomic environment of the unitigs most significantly associated with the tested phenotype. Fig 2 summarises the main steps of the process. A detailed description of the pipeline is presented in the Methods section.

Here we rely on a few experiments to illustrate how the subgraphs output by DBGWAS can be read as genetic events. We then benchmark DBGWAS against two other k-mer-based approaches and one resistome-based approach. DBGWAS recovers known variants, while suggesting novel candidates out of the range of the resistome-based approach. We also find it to be more computationally efficient and to provide more interpretable outputs than the other k-mer-based methods.

A synthetic description of the discussed subgraphs is provided in Table 1, while a description of the top subgraphs obtained for all tested antibiotics is provided in S3, S4, and S5 Tables. The subgraphs themselves are available at http://pbil.univ-lyon1.fr/datasets/DBGWAS_support/experiments/#DBGWAS_all_results.

**Table 1:** Resistance determinants identified by DBGWAS for *S. aureus* (SA), *M. tuberculosis* (TB) and *P. aeruginosa* (PA) panels.

Panel	Phenotype	Rank	Sign. unitigs	*min*_*q*_	Est. effect	Annotation	Type	Knowledge on markers
SA	Methicillin	1	71/565	7.68 × 10^−188^	0.949	*mecA* + 7000 bp of SC *Cmec*	MGE	Pos
		2	99/735	3.39 × 10^−72^	0.865	6000 bp of SCC*mec*	MGE	*r*^2^ = 0.96
		3	11/190	2.14 × 10^−61^	0.813	2000 bp of SCC*mec*	MGE	*r*^2^ = 0.94
		4	13/117	2.29 × 10^−37^	0.957	1500 bp of SCC*mec*	MGE	*r*^2^ = 0.93
	Ciprofloxacin	1	7/57	8.67 × 10^−104^	-0.893	*parC*QRDR	LPG	Pos
		2	7/31	2.21 × 10^−76^	0.955	*gyrA*QRDR	LPG	Pos
	Erythromycin	1	110/510	2.69 × 10^−100^	0.823	*ermC* + circular plasmid	MGE	Pos
	Fusidic acid	1	7/50	2.75 × 10^−136^	-0.910	*fusA*	LPG	Pos
		2	214/882	7.94 × 10^−49^	0.924	*fusC* + SCC *fusC*cassette	MGE	Pos
		3	22/260	5.35 × 10^−43^	0.924	1,500 bp of SCCfusC	MGE	*r*^2^ = 0.98
		3	1/72	5.35 × 10^−43^	0.924	200 bp of SCC*fusC*	MGE	*r*^2^ = 0.98
		5	5/64	2.02 × 10^−22^	-0.888	*purN*	LPG	*r*^2^ = 2 × 10^−3^
	Trimethoprim	1	7/54	8.38 × 10^−24^	0.969	*folA*	LPG	Pos
		2	3/41	9.30 × 10^−18^	-0.966	btw. hyp. prot. & VOC prot.	LPN	*r*^2^ = 0.19
		3	11/70	9.30 × 10^−18^	-0.966	*ybaK*	LPG	*r*^2^ = 0.44
		4	2/30	6.82 × 10^−10^	-0.632	*mqo1*	LPG	*r*^2^ = 0.29
	Gentamicin	1	173/1193	1.30 × 10^−205^	0.873	*aac(6’)*gene within a plasmid	MGE	Pos
		2	127/367	9.02 × 10^−75^	0.751	seq. of plasmid carrying *aac(6’)*	MGE	*r*^2^ = 0.38
		3	2/23	9.01 × 10^−53^	0.634	seq. of plasmid carrying *aac(6’)*	MGE	*r*^2^ = 0.40
		4	1/29	1.04 × 10^−40^	0.579	seq. of plasmid carrying *aac(6’)*	MGE	*r*^2^ = 0.48
		5	2/56	1.49 × 10^−33^	-0.831	*odhB*	LPG	*r*^2^ = 8 × 10^−5^
TB	Rifampicin	1	36/115	4.84 × 10^−70^	-0.577	*rpoB*RRDR	LPG	Pos
		2	6/37	4.35 × 10^−20^	-0.355	*katG*	LPG	CR
		3	5/41	4.02 × 10^−8^	-0.224	*embB*M306V	LPG	Pos
	Streptomycin	1	5/30	3.70 × 10^−31^	0.544	*rpsL*(30S ribos.protein S12)	LPG	Pos
		2	6/37	1.06 × 10^−28^	-0.428	*katG*	LPG	CR
		3	25/113	2.87 × 10^−16^	-0.339	*rpoB*RRDR	LPG	CR
		4	6/45	1.40 × 10^−9^	-0.271	*embB*M306V	LPG	CR
		5	8/31	2.86 × 10^−9^	-0.535	*rrs*, 16S rRNA C517T	LPG	Pos
		6	13/69	9.18 × 10^−5^	-0.216	*gyrA*QRDR	LPG	CR
		7	2/20	1.20 × 10^−3^	0.739	*espG1*	LPG	*r*^2^ = 3 × 10^−3^
	Ofloxacin	1	31/85	9.66 × 10^−144^	-0.888	*gyrA*QRDR	LPG	Pos
		2	9/68	1.59 × 10^−4^	0.507	*ubiA*(Rv3806c)	LPG	CR
		3	3/32	3.86 × 10^−2^	-0.746	Rv3909	LPG	*r*^2^ = 9 × 10^−3^
	Ethionamide	1	9/39	7.86 × 10^−11^	-0.462	*fabG1*promoter	LPN	Pos
		2	15/47	5.16 × 10^−10^	-0.406	*gyrA*QRDR	LPG	CR
		3	4/26	5.55 × 10^−4^	0.319	*rrs*, 16S rRNA A1401G	LPG	CR
	XDR	1	6/68	3.66 × 10^−39^	0.905	*rpoB*I1187T (out. RRDR)	LPG	Ukn
		1	3/27	3.66 × 10^−39^	0.905	Rv2000	LPG	*r*^2^ = 1
		3	3/24	9.58 × 10^−36^	0.883	*espA*promoter	LPN	*r*^2^ = 0.98
PA	Amikacin	1	4/83	5.86 × 10^−9^	0.621	SNP in *aac(6’)*	LPG	Pos
		2	3/82	1.37 × 10^−6^	0.662	DEAD/DEAH box helicase	LPG	*r*^2^ = 0.55
		3	38/315	2.21 × 10^−6^	0.523	plasmid mapping on pHS87b	MGE	*r*^2^ = 0.17
	Levofloxacin	1	5/27	7.21 × 10^−29^	-0.884	*gyrA*QRDR	LPG	Pos
		2	5/29	5.68 × 10^−6^	-0.737	*parC*QRDR	LPG	Pos
		3	5/38	1.87 × 10^−2^	0.688	Histidine kinase/response regulator	LPG	*r*^2^ = 0.17

For each antibiotic, we report subgraphs with their rank, number of significant unitigs over all unitigs in the subgraph (Sign. unitigs), q-value of the unitig with the lowest q-value (min_*q*_), the corresponding estimated effect ($\hat{\beta }$ coefficient of the linear mixed model) and annotation of the subgraph. The type of event represented by the subgraph is colour-coded as: yellow for MGE, light blue for local polymorphism in gene (LPG), and dark blue for local polymorphism in noncoding region (LPN). Known resistance markers are indicated in dark green (Pos), determinants whose presence was described to be caused by co-resistance in orange (CR), unknown variants arriving at the first rank in grey (Ukn). For other subgraphs, an *r*^2^ value relative to the first subgraph is provided as an estimation of linkage disequilibrium with the first subgraph. It was computed between the most significant patterns of the first and the considered subgraphs.

### Coloured bubbles highlight local polymorphism in core genes, accessory genes and noncoding regions

**Fig 3:**
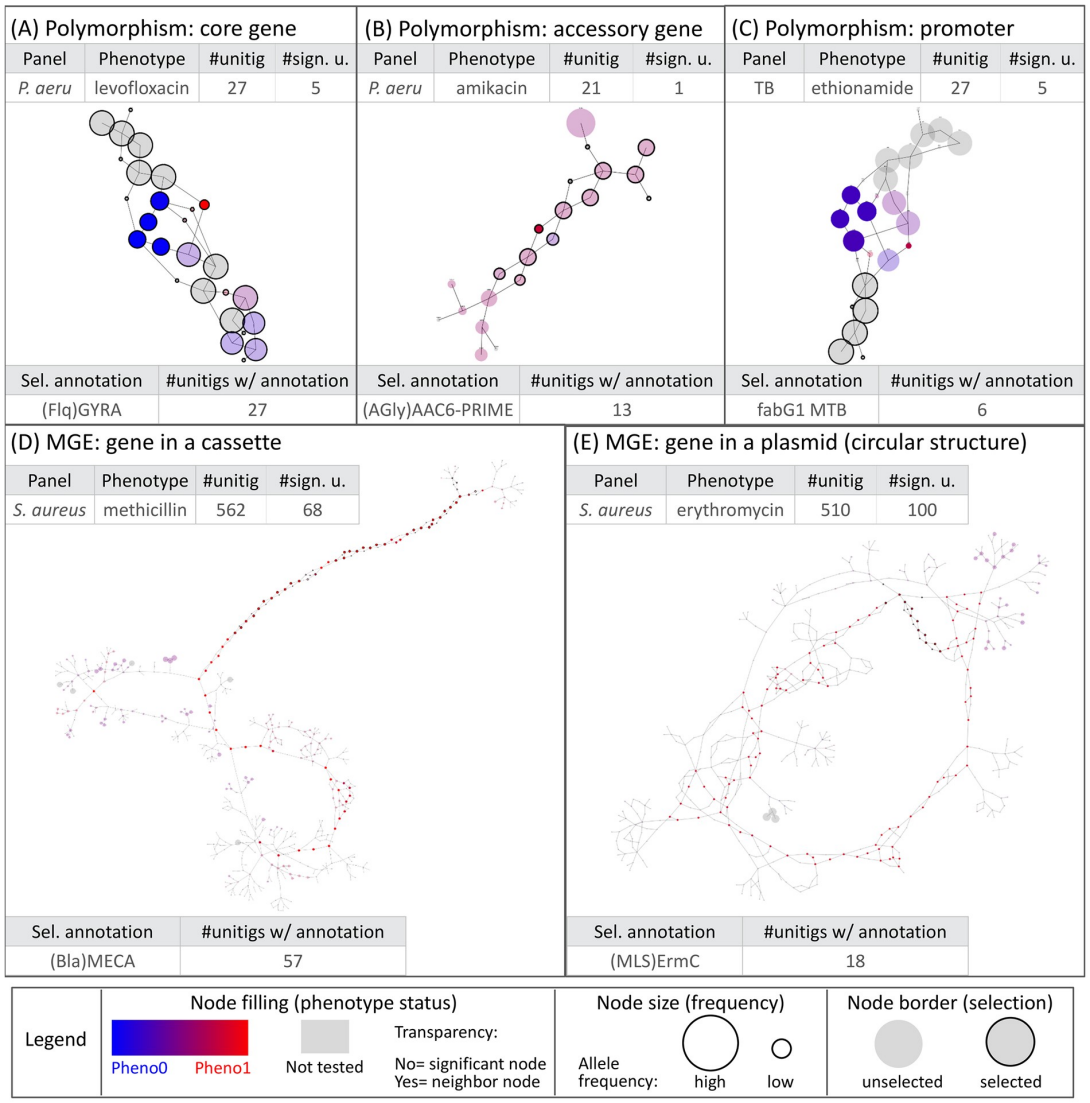
Different types of genetic events identified by DBGWAS. Each subgraph represents a distinct genetic event. Colours are continuously interpolated between blue for susceptible unitigs and red for resistant ones. Untested unitigs, present in > 99% or < 1% of the strains, are shown in grey. Nodes found to be not significative are shown with a transparency degree. The node size relates to its allele frequency: the larger the node, the higher the allele frequency. Circled black nodes map to annotated genes. The two tables in each panel provide information on the sugraph nodes. As an example, the subgraph in panel (A) is composed of 27 unitigs, 5 of which were significantly associated with resistance. All unitigs of this subgraph mapped to the *gyrA* gene. The subgraphs presented in the four other panels correspond to the top subgraphs (with lowest min_*q*_) obtained for different panels/phenotypes. All subgraphs are snapshots taken from DBGWAS interactive visualisation and are available online.

For *P. aeruginosa* levofloxacin resistance, the subgraph obtained with the lowest min_*q*_ highlighted a polymorphic region in a core gene (Fig 3A). Indeed, it showed a linear structure containing a complex bubble, with a fork separating susceptible (blue) and resistant (red) strains. The annotation revealed that all unitigs in this subgraph mapped to the quinolone resistance-determining region (QRDR) of the *gyrA* gene. *gyrA* codes for a subunit of the DNA gyrase targeted by quinolone antibiotics such as levofloxacin and its alteration is therefore a prevalent and efficient mechanism of resistance [20, 21]. In all our experiments related to quinolone resistance, DBGWAS identified QRDR mutations in either *gyrA* or *parC*, which codes for another well-known quinolone target: *P. aeruginosa* levofloxacin (first subgraph, *gyrA*: min_*q*_ = 7.21 × 10^−29^ and second, *parC*: 5.68 × 10^−06^), *S. aureus* ciprofloxacin (first, *parC*: min_*q*_ = 8.67 × 10^−104^ and second, *gyrA*: 2.21 × 10^−76^), and ofloxacin resistance in *M. tuberculosis*, whose genome does not contain the *parC* gene [22] (first, *gyrA*: min_*q*_ = 9.66 × 10^−144^).

For *P. aeruginosa* amikacin resistance, the top subgraph (min_*q*_ = 5.86 × 10^−9^) highlighted a SNP in an accessory gene (Fig 3B). As in Fig 3A, it contained a fork separating a blue and a red node. However, other remaining nodes were not grey: they represented an accessory sequence because they were not present in all the strains. Most of these nodes were pale-red, showing that the accessory sequence was more frequent in resistant samples. The annotation revealed that this subgraph corresponded to *aac(6’)*, a gene coding for an aminoglycoside 6-acetyltransferase, an enzyme capable of inactivating aminoglycosides, such as amikacin, by acetylation [23]. Most unitigs in this gene had a low association with resistance, except for the ones describing this particular SNP. Mapping the sequence of these unitigs on the UniProt database [24] revealed an amino-acid change at L83S, right in the enzyme binding site. This SNP was previously shown to be responsible for substrate specificity alteration in a strain of *Pseudomonas fluorescens* [25]. It appears to increase the amikacin acetylation ability of *aac(6’)*, making its association to amikacin resistance more significant than the gene presence itself.

Finally, for *M. tuberculosis* ethionamide resistance, the top subgraph (min_*q*_ = 7.86 × 10^−11^, Fig 3C) represented a polymorphic region in a core gene promoter. The subgraph was mostly grey and linear with a localised blue and red fork. The most reliable annotation for this subgraph was *fabG1* (also known as *mabA*), a core gene previously shown to be involved in ethionamide and isoniazid resistance [26, 27]. None of the significantly associated unitigs mapped to the *fabG1* gene, but their close neighbours did (highlighted in Fig 3C by black circles), suggesting that the detected variant was located in the promoter region of the gene. This was confirmed by mapping the significant unitig sequences using the Tuberculosis Mutation database of the *mubii* resource [28].

### Long single-coloured paths denote mobile genetic element insertions

For *S. aureus* resistance to methicillin, the top subgraph (min_*q*_ = 7.68 × 10^−188^), shown in Fig 3D, revealed a gene cassette insertion. It contained a long path of red nodes, and a branching region including another red node path. The first path mapped to the *mecA* gene, extensively described in this context and known to be carried by the Staphylococcal Cassette Chromosome *mec* (SCC*mec*) [21, 29, 30]. The other part of the subgraph represented a >5,000 bp fragment of the cassette. It was less linear because it summarised several types of the cassette differing by their structure and gene content [29]. The next subgraphs represented other regions of the same cassette. Interestingly, retaining a greater number of unitigs to build the subgraphs leads to merging these individual subgraphs, representing related genomic regions, into a single one. This can be done by increasing the Significant Features Filter (*SFF*) parameter value, which defines the unitigs used to build the subgraphs. By default, the unitigs corresponding to the 100 lowest q-values are retained (*SFF* = 100). Increasing the *SFF* value to 150 (150th q-value = 1.60 × 10^−27^) allowed us to reconstruct the entire SCC*mec* cassette, as shown in S3 Fig.

For *S. aureus* erythromycin resistance, a unique subgraph was generated (min_*q*_ = 2.69 × 10^−100^). As shown in Fig 3E, the subgraph described the circular structure of a 2,500 bp-long plasmid known to carry the causal *ermC* gene together with a replication and maintenance protein in strong linkage disequilibrium with *ermC* [30, 31].

For *P. aeruginosa* amikacin resistance, the third subgraph (min_*q*_ = 2.21 × 10^−6^) represented a 10,000 bp plasmid acquisition. Using the NCBI nucleotide database [32], most of the unitigs in this subgraph mapped to the predicted prophage regions of an integrative and conjugative plasmid, whose structure corresponds to a plasmid, pHS87b, recently described in the amikacin resistant *P. aeruginosa* HS87 strain [33]. S4 and S5 Figs provide more examples of MGEs recovered by DBGWAS, and the Interpretation of significant unitigs (step 3) subsection of the Methods section discusses *SFF* default value and tuning.

### DBGWAS reports expected variants without prior knowledge

Although resistance determinants are not perfectly or exhaustively known for all species, some resistance mechanisms are well described. This is the case of *gyrA* and *parC* alteration in fluoroquinolone resistance in *P. aeruginosa* [20], and of the alteration of two streptomycin targets: the ribosomal protein S12 (coded by *rpsL*) and the 16S rRNA (coded by *rrs*) in *M. tuberculosis* [34]. Here we verify the ability of bacterial GWAS methods to recover these known mechanisms. We compared DBGWAS results to those obtained by applying the same association model to a collection of known resistance genes and SNPs [7, 35] (see the Resistome-based association studies subsection of the Methods section), and to two other recent k-mer-based methods: pyseer [6, 36], and HAWK [13].

For *P. aeruginosa* levofloxacin resistance (Table 2), both DBGWAS and pyseer identified the two expected known causal determinants reported by the prior resistome-based study: *gyrA* and *parC*, while HAWK only reported *gyrA*. pyseer reported 224 k-mers, all mapping to *gyrA* and *parC*, while the other methods reported less than 10 features (subgraphs or reassembled k-mers), among which were several unknown, potentially new candidate markers.

**Table 2:** Resistance determinants found by the four methods for *P. aeruginosa* levofloxacin resistance.

**Legend**	resistome-based	DBGWAS	pyseer	HAWK
Time (mem)	37m (7.2 GB)	21m (3.2 GB)	24h22m (14.5 GB)	39m (4.2 GB)
Nb reported	2 variants	5 subgraphs	224 k-mers	8 reassembled k-mers
Known positive	*gyrA* (2.11 × 10^−22^)	*gyrA* (7.21 × 10^−29^)	*gyrA* (1.97 × 10^−17^)	*gyrA* (2.82 × 10^−14^)
	*parC* (1.83 × 10^−5^)	*parC* (5.68 × 10^−6^)	*parC* (5.68 × 10^−9^)	
Unknown		HK/RR (1.87 × 10^−2^)		tnp (1.66 × 10^−14^)
		tnp		NC near tnp
		*topA*		

This table presents the annotation of the features identified by the tested methods with default parameters. The total number of reported features, as well as the execution time and memory load (in Gigabytes) are given in the header. For k-mer-based methods, annotations were retrieved by mapping unitig/k-mer sequences to the resistance and Uniprot databases (see Interpretation of significant unitigs (step 3) subsection of the Methods section), and completed when needed by Blast on NCBI Nucleotide database. Green cells correspond to resistance determinants already described in the literature. Grey cells represent unknown determinants. Within each category, annotations are ordered by increasing minimum p/q-values. p/q-values are reported only for the most significant annotations. For each method, the annotation with the lowest p/q-values is underlined. ‘NC’ means noncoding region and ‘tnp’ transposase.

For *M. tuberculosis* streptomycin resistance (Table 3), the four methods reported the two expected known causal determinants *rpsL* and *rrs*. However, while the resistome-based study and DBGWAS methods ranked the causal *rpsL* determinant first, pyseer and HAWK reported their lowest p/q-values for the false positive *katG* determinant. *katG* and other false positives caused by co-resistance were among the top-ranked features for all methods and this is a well described phenomenon in *M. tuberculosis* species [34, 37].

**Table 3:** Resistance determinants found by the four methods for *M. tuberculosis* streptomycin resistance.

**Legend**	resistome-based	DBGWAS	pyseer	HAWK
Time (mem)	1h31m (2.1 GB)	42m (4.3 GB)	14h14m (102.4 GB)	3h01m (3.7 GB)
Nb reported	28 variants	24 subgraphs	85,011 k-mers	2,038 reassembled k-mers
Known positive	*rpsL* (1.96 × 10^−33^)	*rpsL* (3.70 × 10^−31^)	*rpsL* (4.85 × 10^−55^)	*rpsL* (5.72 × 10^−47^)
	*rrs* (5.40 × 10^−8^)	*rrs* (2.86 × 10^−9^)	*rrs* (1.63 × 10^−14^)	*rrs* (3.45 × 10^−20^)
Determinant described for other antibiotics	*katG* (2.61 × 10^−30^)	*katG* (1.06 × 10^−28^)	*katG* (2.12 × 10^−71^)	*katG* (1.44 × 10^−57^)
	*rpoB*	*rpoB*	*rpoB*	*embB*
	*gidB*	*embB*	*embB*	***kasA***
	*gyrA*	*gyrA*	***ubiA***	***embC***
	*embB*	*gidB*	*pncA*	*gyrA*
	*fabG1* promoter	*rpoC*	*fabG1* promoter	***iniA***
	*pncA*	*fabG1* promoter	*gyrA*	***embA***
	*rpoC*	***ubiA***	*gidB*	***embR***
	*inhA*		***ethA***	*gidB*
			***embA***	***tsnR***
			***embC***	*rpoB*
				*pncA*
				***ethA***
Unknown (top list)		*espG1* (1.20 × 10^−3^)	NC near tnp/PE (1.13 × 10^−19^)	NC near tnp/PPE (2.93 × 10^−57^)
		*rpsN*	Rv0270	tnp
		NC near tnp/PPE	Rv2665	Rv2825c/Rv2828c
		*rnj*	Rv2743c	13E12 repeat family protein
		Rv2672	Rv2522c	PPE
		*espA* promoter	NC near tnp/PPE	CRISPR repeats, down *Cas* genes
		Rv2456c promoter	*guaA*	*mmpL14*
		*whiB6*	*kdpD*	*esxM*
		…	…	…

This table presents the annotation of the features identified by the tested methods with default parameters. The total number of reported features, as well as the execution time and memory load (in Gigabytes) are given in the header. For k-mer-based methods, annotations were retrieved by mapping unitig/k-mer sequences to the resistance and Uniprot databases (see Interpretation of significant unitigs (step 3) subsection of the Methods section), and completed when needed by Blast on NCBI Nucleotide database. Green cells correspond to resistance determinants already described in the literature, orange cells to resistance determinants described for association with other antibiotics. The annotations not found by the resistome-based strategy are written in bold. Grey cells represent unknown determinants. Within each category, annotations are ordered by increasing minimum p/q-values. p/q-values are reported only for the most significant annotations. For each method, the annotation with the lowest p/q-values is underlined. ‘NC’ means noncoding region, ‘tnp’ transposase, ‘PE’ stands for PE-family protein and ‘PPE’ for PPE-family protein.

Additional results for all antibiotics can be found in S6 and S7 Tables for resistome-based association studies, and in S3 and S5 Tables for DBGWAS.

### DBGWAS provides novel hypotheses

In addition to resistance markers, all three k-mer-based approaches reported several unknown variants, not described in the context of resistance. Among them, in the context of streptomycin resistance, a noncoding region between a transposase and a PPE-family protein was reported by the three methods but, as expected, not by the resistome-based approach, as only resistance genes were included in this analysis. More generally, knowledge-based approaches such as SNP-, gene- or resistome-based GWAS can be limited in the context of new marker discovery, since any causal variant absent from the chosen reference would remain untested. Besides being time-consuming, preparing such a list of genetic variants can be problematic for bacterial species without extensive annotation or reference availability. Here we describe associations identified by DBGWAS and which were never described in the antibiotic resistance literature.

In our *P. aeruginosa* panel, the second subgraph obtained for amikacin resistance (min_*q*_ = 1.37 × 10^−6^) gathered unitigs mapping to the 3’ region of a DEAD/DEAH box helicase, known to be involved in stress tolerance in *P. aeruginosa* [38]. The unitig with the lowest q-value was present in 13 of 47 resistant strains and in only 1 of 233 susceptible strains and represented a C-C haplotype summarising two mutated positions: 2097 and 2103. This annotation was not an artefact of the population structure, properly taken into account by the linear mixed model. Indeed the 13 resistant strains corresponded to distinct clones belonging to two phylogroups, one of them containing the susceptible strain. In *P. aeruginosa* levofloxacin resistance, the third subgraph (min_*q*_ = 1.87 × 10^−2^) represented a L650M amino-acid change in a hybrid sensor histidine kinase/response regulator. Such two-components regulatory systems play important roles in the adaptation of organisms to their environment, for instance in the regulation of biofilm formation in *P. aeruginosa* [39], and as such may play a role in antibiotic resistance.

In *S. aureus*, polymorphisms within genes not known to be related to resistance were identified for several antibiotics: *purN* (min_*q*_ = 2.02 × 10^−22^) for fusidic acid, *odhB* (min_*q*_ = 1.49 × 10^−33^) for gentamicin, *ybaK* and *mqo1* (min_*q*_ = 9.30 × 10^−18^, resp. 6.82 × 10^−10^) for trimethoprim. None of these genes have been associated with antibiotic resistance before, to the best of our knowledge.

In *M. tuberculosis*, polymorphisms in two genes encoding proteins involved in *cell wall and cell processes*, *espG1* and *espA*, were found associated with streptomycin (seventh subgraph, min_*q*_ = 9.43 × 10^−4^) and XDR phenotype (third subgraph, min_*q*_ = 9.58 × 10^−36^), respectively. Again, these genes have never been reported in association with antibiotic resistance before.

Although experimental validation would be required to tell whether these hypotheses are false positive (e.g., in linkage with causal variants) or actual resistance mechanisms not yet documented, DBGWAS is a valuable tool to screen for novel candidate markers. Moreover it provides a first level of variant description (SNPs in gene or promoter, MGE, etc) which can directly drive the biological validation.

### DBGWAS facilitates the interpretation of k-mer-based GWAS

Other k-mer-based approaches are as agnostic as DBGWAS and were also able to provide novel hypotheses, but interpreting their output can prove more challenging than a SNP/gene-based GWAS. In the *M. tuberculosis* streptomycin resistance experiment for example, they reported several thousands of features, while DBGWAS reported only 24 annotated subgraphs without missing any expected determinant (see Table 3). The thousands of k-mers generated by HAWK and pyseer are of course also amenable to interpretation: to build our Table 3, we mapped these k-mers to references and extracted annotated variants which showed at least one hit. However, doing so required additional efforts and a working knowledge of the most appropriate annotated references. In addition, k-mers which do not map to the chosen reference cannot be interpreted. By contrast, DBGWAS always returns a subgraph containing these k-mers. Even when no annotation exists, the topology and colours of the subgraphs may hint towards the nature of the causal variant.

In addition to providing context for significant k-mers and guiding their interpretation as SNPs or MGEs, DBGWAS clustering of close variants into a subgraph can describe hypervariable regions as single entities, and highlight highly associated haplotypes. As an example, the top subgraph for rifampicin resistance (min_*q*_ = 4.84 × 10^−70^) contained 36 significant unitigs, distinguishing between susceptible (blue) and resistant (red) strains. Instead of a single point mutation, this subgraph represented a polymorphic region known as the rifampicin resistance-determining region (RRDR) of the *rpoB* gene. The unitig with the lowest q-value covered several mutant positions, defining a particular haplotype strongly associated with rifampicin susceptibility. Where DBGWAS reported in this case only one subgraph, pyseer, for instance, reported 470 k-mers with the *rpoB* annotation, and the resistome-based association study reported in this case 4 distinct SNPs in *rpoB* (S6 Table). In another user-submitted example, DBGWAS identified mosaic alleles of three *pbp* genes involved in beta-lactam resistance of *Streptococcus pneumoniae*. Like in the RRDR example, it returned five subgraphs corresponding to the three genes—three subgraphs were annotated *pbp2x* and represented three distinct polymorphic regions of the gene. Each subgraph summarised the polymorphism of the gene, as opposed to one separate feature for each SNP.

Admittedly, some subgraphs output by DBGWAS are not readily interpretable: they are neither coloured bubbles highlighting SNPs, nor long single-coloured paths denoting MGE insertions. This was the case of several subgraphs produced for *P. aeruginosa* amikacin resistance, and presented in S6 Fig. Genetic variants inserted in variable regions, for example, lead to subgraphs with a high average degree, or to very large subgraphs. The fourth subgraph for instance (min_*q*_ = 2.21 × 10^−6^) contains a path of three red (positively-associated) nodes lying in a noncoding region between variable accessory genes. Consequently, their neighbour unitigs branch to various other unitigs, making the structure complex and hard to interpret. Complex subgraphs also arise when several associated variants have overlapping neighbourhoods (as defined in the Graph neighbourhoods subsection in the Methods section, and tuned with the *nh* parameter) in at least one strain. This is the case for the subgraph with the smallest min_*q*_ which aggregates *aac*(6′) acetyltransferase and the CML efflux pump.

The interpretation of such subgraphs is not straightforward. We often found it helpful to tune the *nh* and *SFF* parameters to break large subgraphs into a set of smaller ones, as discussed in the discussed in the Methods section. For the *aac*(6′) subgraph, where nearby variants are aggregated into a large subgraph, reducing the *SFF* value to 15 provided a much smaller and easier-to-interpret subgraph focusing on the *aac*(6′) mutation (Fig 3B). Otherwise, we recommend to focus on the topology of the most significant unitigs and their close neighbours.

### DBGWAS is fast, memory-efficient, and scales to very large panels

To assess the scalability of DBGWAS to large datasets, we retrieved 5,000 genomes from *M. tuberculosis*, 9,000 genomes from *S. aureus* and 2,500 genomes from *P. aeruginosa*, as described in the Large panels subsection of the Methods section. We present in S9 Fig the runtime and memory usage performances for these panels. All 180 runs took less than 5 days and 250 GB of RAM on 8 cores. Both the computational time and memory usage increase log-linearly with the panel size. Moreover, at equal panel size, DBGWAS performance also depends on the genome complexity, requiring less computational resource for more clonal genomes such as *M. tuberculosis*.

We also compared the computational performance of DBGWAS with pyseer and HAWK. The benchmark was performed on 13 datasets, including one large dataset of 2,500 genomes for each of the 3 species (see the Datasets subsection in the Methods section for details). Detailed results are presented in S2 Table. DBGWAS was the fastest tool in 11 out of 13 experiments, always taking less than 2 hours. HAWK ran in less than 10 hours in 12 out of 13 experiments, and was a little faster than DBGWAS on two of the large-scale datasets. pyseer took from 13 to 53 hours on 9 experiments, and failed on the 4 others: one exceeded the disk space limit of 1TB, three exceeded the runtime limit of five days. It was brought to our attention during the reviewing process that piping the output of fsm-lite through gzip would decrease the disk space usage. HAWK was more parsimonious in memory usage than DBGWAS on the large scale panels. This can be explained by the fact that the 0.8.3-beta version of HAWK which we are using does not take into account the population structure, and as such does not have to compute an *n* × *n* covariance matrix, providing it a large gain in memory usage—and, to a lesser extent, runtime—for large panels. On the other hand, disregarding the population structure could also lead to spurious discoveries. HAWK v0.9.8-beta offers an adjustment but failed to recover the known true positives, which is why we chose to present the results of the 0.8.3-beta version. DBGWAS and HAWK typically used one order of magnitude less memory than pyseer. The most memory-consuming step for pyseer was the k-mer counting step relying on fsm-lite.

## Discussion

In this article we introduce an efficient method for bacterial GWAS. Our method is agnostic: it considers all regions of the genomes and is able to identify potentially new causal variants as different as SNPs in noncoding regions and MGE insertions/deletions. It performs as well as the current SNP- and gene-based gold standard approaches for retrieving known determinants, from genome pre-assemblies and without relying on annotations or reference genomes.

DBGWAS exploits the genetic environment of the significant k-mers through their neighbourhood in the cDBG, providing a valuable interpretation framework. Because it uses only contig sequences as input, it allows GWAS on bacterial species for which the genomes are still poorly annotated or lack a suitable reference genome. DBGWAS makes bacterial GWAS possible in two hours using a single-core computer (see S1 Table), outperforming other state-of-the-art k-mer-based approaches.

Underlying our method, graph-based genome sequence representations such as DBGs, extend the notion of the reference genome to cases where a single sequence stops being an appropriate approximation [40, 41]. As demonstrated in this paper, they pave the way to GWAS on highly plastic bacterial genomes and could also be useful for microbiomes [42] or human tumours [13].

DBGWAS currently relies on the Benjamini-Hochberg procedure to control the FDR and offers no advance exploiting the dependence among presence/absence patterns. An important improvement would be to control the false discovery rate at the subgraph level instead of the unitig level. DBGWAS could be extended to different statistical tasks by adapting its underlying association model, to allow for continuous phenotypes or identify epistatic effects, for instance. The interpretability of the extracted subgraphs could also be improved by training a machine learning model to predict which types of event they represent [43]. This automated labelling could guide users in their interpretation and allow them to search for specific events, such as SNPs in core genes or rearrangements.

Several recent studies describe *in silico* models for defining a genomic antibiogram and hopes are high that such technologies will complement the classic phenotypic methods [44]. Several studies have already demonstrated that in some cases, genomic antibiograms can be at least as good as phenotypic ones [30, 45–47]. Contrary to our approach, these studies require extensive resistance marker databases. DBGWAS will surely contribute to the extension of such databases or to the development of agnostic genomic antibiograms.

In conclusion, we demonstrate for three medically important bacterial species that resistance markers can be detected rapidly with relative ease, using simple computer equipment. Our integrated software and visualisation tools offer an intuitive variant representation, hence will provide future users with an enhanced insight into genotype to phenotype correlations, in all domains of microbiology, beyond that of antibiotic resistance. This will include complex traits such as biofilm formation, epidemicity and virulence.

## Methods

### Encoding genomic variation with compacted DBGs

DBGs are directed graphs that efficiently represent all the information contained in a set of sequences. Nodes represent all the unique k-mers (genome sequence substrings of length *k*) extracted from the input sequences. Edges represent (*k* − 1)-exact-overlaps between k-mers: an edge connects a node *n*_1_ to a node *n*_2_ if and only if the (*k* − 1)-length-suffix of *n*_1_ equals the (*k* − 1)-length-prefix of *n*_2_ (Fig 1A).

These graphs can be compacted into cDBGs by merging linear paths (sequences of nodes not linked to more than two other nodes) into a single node referred to as a *unitig* [48–50] (Fig 1C). Compaction yields a graph with locally optimal resolution: regions of the genome which are conserved across individuals are represented by long unitigs, while regions which are highly variable are fractioned into shorter unitigs (S1 Fig).

### Representing strains by their unitig content (step 1)

#### cDBG construction

We build a single DBG from all genomes given as input using the GATB C++ library [51]. We start from contigs rather than reads and, consequently, we do not need to filter out low abundance k-mers, allowing for the exploration of any variation present in the set of input genomes. We then compact the DBG using a graph traversal algorithm, which identifies all linear paths in the DBG—each forming a unitig in the cDBG. During this step, we also associate each k-mer index to its corresponding unitig index in the cDBG.

**Fig 4:**
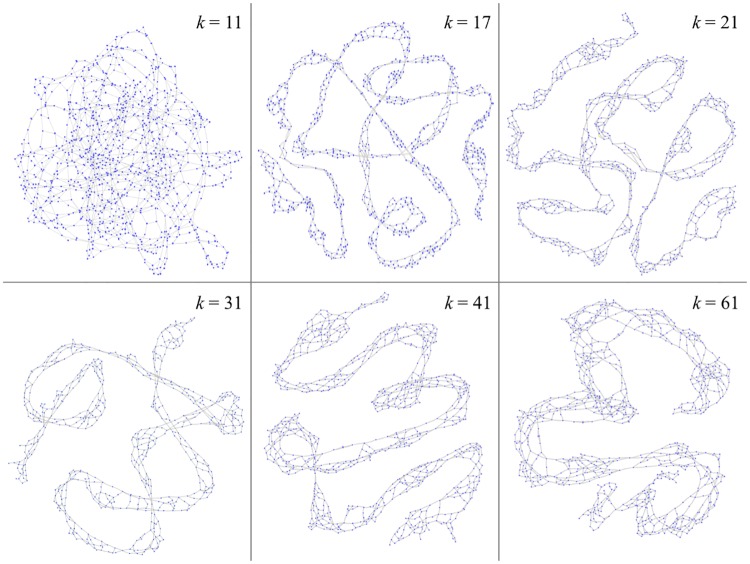
Effect of *k* on the graph topology. A cDBG was built from the *P. aeruginosa gyrA* gene sequences from several strains. When *k* is small, k-mers are highly repeated, which generate numerous loops. As *k* increases, k-mer sequences become more specific and the graph gets more linear. For large values of *k*, few k-mers are shared by all the strains, and the linear path thickens into parallel paths belonging to variable strain populations.

**Fig 5:**
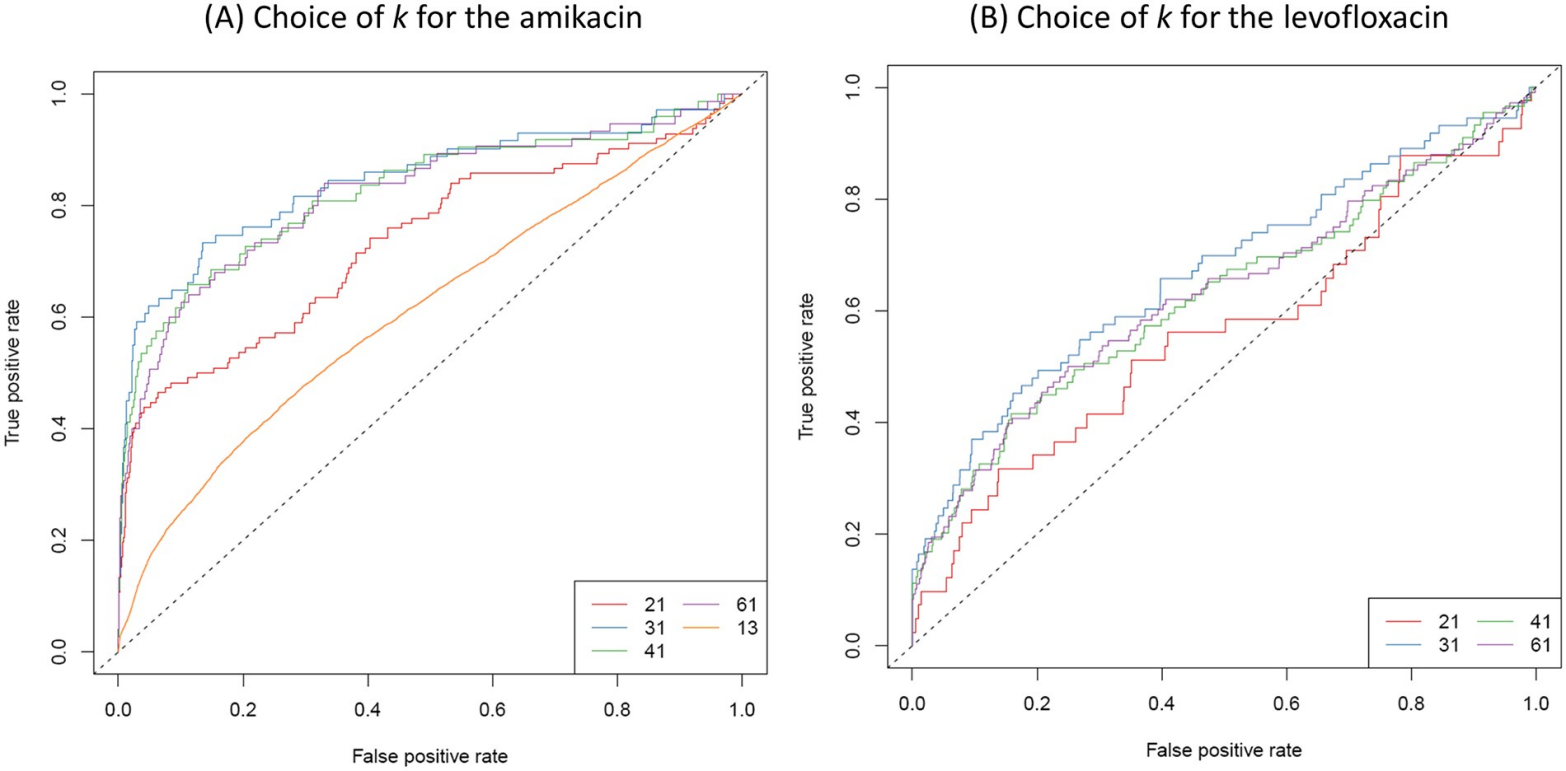
Choice of *k*. True positive *versus* false positive curves for several values of *k* for both amikacin and levofloxacin resistance phenotypes. True positives are unitigs mapping to genuine variants described in resistance databases for the studied drugs [7]. In both cases, the value of *k* leading to the best AUC is *k* = 31.

There is no general rule for choosing the ideal k-mer length as it depends on many factors, including the assembly quality, complexity of the input genomes, or presence of repeats. High values of *k* lead to haplotypes containing multiple SNPs instead of distinct single SNPs, if these SNPs are separated by less than *k* bases. As *k* increases, the k-mer-defined haplotypes also become more specific to a genome sub-population, leading to a loss of power to detect genotype to phenotype associations. Low values of *k*, on the other hand, produce highly connected sets of non-specific k-mers. In particular, any repeated region with at least *k* bases may create a cycle in the DBG (Fig 4). We use *k* = 31 by default, as it produced the best performance to retrieve known markers of *P. aeruginosa* resistance to amikacin and levofloxacin (Fig 5). We found DBGWAS results to be robust to small variations of *k* between 21 and 41. Similar graph structures were generated whatever the tested value of *k* for the clonal *M. tuberculosis* species (S7 Fig). More variability was observed for *P. aeruginosa* resistance to amikacin, which involves more complex resistance mechanisms (S8 Fig).

#### Unitig presence across genomes

Each genome is represented by a vector of presence/absence of each unitig in the cDBG. To do so, we query the unitig associated to each k-mer in a given genome. This procedure is efficient because it relies on constant time operations. Firstly, we use GATB’s Minimal Perfect Hash Function (MPHF) [52] to retrieve the index of a given k-mer, and then we use the previously computed association between k-mer and unitig indices to know which unitigs the given genome contains. Since these two operations take constant time, producing this vector representation for a genome takes linear time on the size of the genome. It is important to note that the GATB’s MPHF can be successfully applied here because we always use the same list of k-mers, i.e., after building the DBG, the set of k-mers is fixed and not updated, and because we always query k-mers that are guaranteed to be in the DBG (since we do not filter out any k-mer).

The unitig description on all the input genomes is stored into a matrix *U*:

$$U_{i,j}=\{\begin{array}{c} 1\text{,}\;\text{if}\;\text{the}\;j\text{-th}\;\text{unitig}\;\text{is}\;\text{present}\;\text{in}\;\text{the}\;i-\text{th}\;\text{input}\;\text{genome};\\ 0\text{,}\;\text{otherwise.} \end{array}$$

We then transform the matrix *U* into *Z*, which represents the minor allele description, in terms of presence [5]: *Z* is identical to *U* except for columns with a mean larger than 0.5, which are complemented: *Z*_*j*_ = 1 − *U*_*j*_ for these columns.

We then restrict *Z* to its set of unique columns. If several unitigs have the same minor allele presence pattern, then they will be represented by a single column. Keeping duplicates would lead to performing the same statistical test several times. Finally, we filter out columns whose average is below 0.01—the user can specify this threshold using the -maf option. We denote the de-duplicated, filtered matrix of patterns by *X*.

Importantly, both k-mers and unitigs lead to the same set of distinct patterns across the genomes. Indeed, every unitig represents (at least) one k-mer, and conversely every k-mer is represented by one (single) unitig. When de-duplicated, the two representations therefore lead to the same set of patterns to be tested for association with the phenotype.

### Testing unitigs for association with the phenotype (step 2)

Human GWAS literature extensively discusses how testing procedures can result in spurious associations if the effect of the population structure is not taken into account [53–55]. Population structures can be strong in bacteria because of their clonality [5, 6, 56, 57]. An additional performance analysis comparing several models for population structure, on both simulated and real data, showed that correcting for population structure using LMMs is often preferable to using a fixed effect correction or not correcting at all (S1 Appendix).

We thus rely on the bugwas method [5], which uses the linear mixed model (LMM) implemented in the GEMMA library [58], to test for association with phenotypes while correcting for the population structure. This method also offers the possibility to test for lineage effects, by calculating p-values for association between the columns of the matrix representing the population structure, and the phenotype [5]. DBGWAS optionally provides bugwas lineage effect plots when the user specifies a phylogenetic tree using the -newick option. An example of the generated figures is available at http://pbil.univ-lyon1.fr/datasets/DBGWAS_support/full_dataset_visualization/.

Formally, the LMM represents the distribution of the binarized phenotype *Y*_*i*_, given the *j*-th minor allele pattern *X*_*ij*_ and the population structure represented by a set of factors $W\in R^{n\leq p}$, by:

$$Y_{i}=X_{ij}\beta +W_{i}^{T}\alpha +\varepsilon_{ij},\;j=1,\ldots ,p.$$ (1)

*β* is the fixed effect of the tested candidate on the phenotype, $\alpha ∼N(0,\sigma_{a}^{2})$, $\sigma_{a}^{2}>0$ is the random effect of the population structure, and $\varepsilon_{ij}\overset{\text{iid}}{∼}N\left(0,\sigma^{2}\right)$ are the residuals with variance *σ*^2^ > 0. *W* is estimated from the *Z* matrix, which includes duplicate columns representing both core and accessory genome. More precisely, denoting *Z* = *USV*^⊤^ the singular value decomposition of *Z*, we use *W* = *US*.

We test *H*_0_: *β* = 0 versus *H*_1_: *β* ≠ 0 in Eq 1 for each pattern using a likelihood ratio procedure producing p-values and maximum likelihood estimates $\hat{\beta }$. To tackle the situation of multiple testing caused by the high number of tested patterns, we compute q-values, which are the Benjamini-Hochberg transformed p-values controlling for false discovery rate (FDR) [59].

### Interpretation of significant unitigs (step 3)

The LMM is used to identify de-duplicated minor allele presence patterns significantly associated with the phenotype at a chosen FDR level. While the testing step is done at the pattern level, the interpretation of the selected features is done at the unitig level. As a result of the de-duplication procedure, a given pattern may correspond to several distinct unitigs. To faithfully interpret the results, all the unitigs corresponding to the significant patterns are retrieved and are assigned the q-value of their pattern. We now show how the initial cDBG can be used in the interpretation step.

#### Significance threshold

The interpretation step focuses on the unitigs with the lowest q-values. These unitigs are indeed used to build the resulting annotated subgraphs. The unitig selection can be either based on the FDR (q-value threshold) or on a number of presence/absence patterns ordered by increasing q-values. Practically, this is done in DBGWAS using a Significant Features Filter (SFF). For a selection based on a FDR threshold, the SFF value is set between 0 and 1, while any integer value > 1 defines the number of patterns to consider.

In our experiments, we choose not to apply a fixed FDR threshold, even though DBGWAS offers this option. Different datasets lead to different q-values, even by several orders of magnitude, and a single FDR threshold would lead to selecting a large number of unitigs generating more than 1,000 subgraphs on some of them (e.g. *S. aureus* ciprofloxacin) as shown in S8 Table. Instead, we retain the 100 patterns with lowest q-values. Although arbitrary, this choice is tractable for all datasets and provides satisfactory results in our experiments. It does not provide and explicit control of the FDR: only the q-value provides an estimation of the proportion of false discoveries incurred when considering patterns below this value. Checking the q-values of the selected unitigs is therefore essential to assess their significance. If the default SFF = 100 is not satisfactory, it is also possible to re-run the third step only, with a more suitable SFF value.

#### Graph neighbourhoods

**Fig 6:**
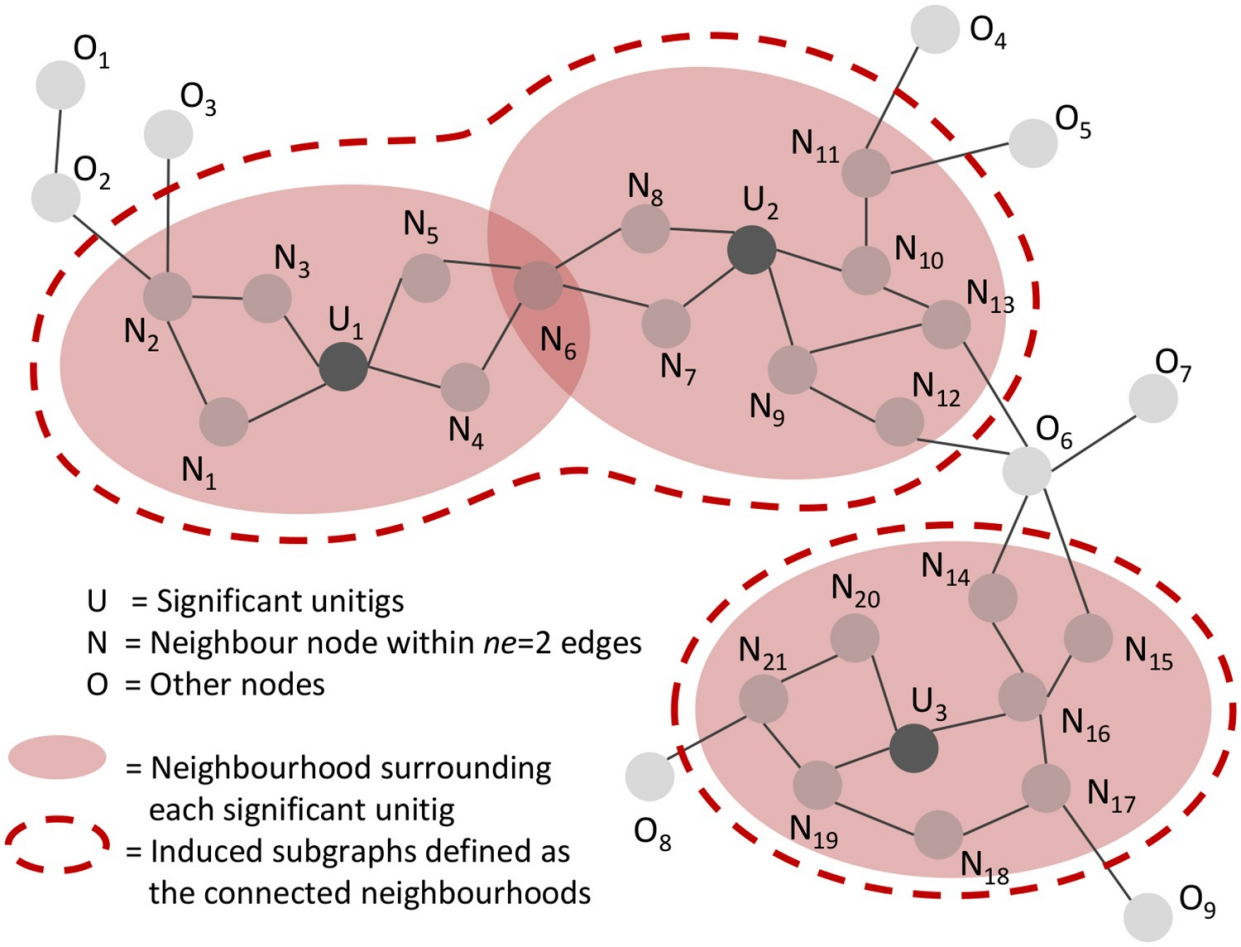
Subgraphs induced by the neighbourhood of significantly associated unitigs. In this example, a neighbourhood of size *ne* = 2 was used: any unitig distant up to 2 edges from a significant unitig is retrieved to define its neighbourhood. Neighbourhoods are merged if they share at least one node, e.g. the neighbourhoods of *U*_1_ and *U*_2_ are merged because they share *N*_6_, and will be represented in a single subgraph.

We define the neighbourhood of each significant unitig *u* (defined by the *SFF*) as the set of unitigs whose shortest path to *u* has at most *ne* = 5 edges. Users can modify the *ne* value using the -nh option. The objects returned by DBGWAS are the connected components of the graph induced by the neighbourhoods of all significant unitigs in the cDBG. As illustrated in Fig 6, nearby significant unitigs might belong to the same connected component, so this process groups unitigs which are likely to be located closely in the genomes. We refer to the connected components as *subgraphs* in the Results section.

The *SFF* value can be tuned to optimise the number and size of the output subgraphs. It has no impact on subgraphs describing SNPs in core sequences (S2 Fig). On the other hand, when significant unitigs map to different regions of a single MGE, such as a plasmid, several subgraphs are generated but can be gathered into a single subgraph by increasing the *SFF* threshold (S4 Fig). When significant unitigs map to several distinct mobile regions, which can be found in different contexts (transposon, integron, etc.) at the population level, the resulting subgraph can become very large and highly branching: decreasing the *SFF* threshold allows to select the few most significant unitigs, generating a subgraph focusing on the most relevant region (S6 Fig). Reducing the graph complexity can also be done by decreasing the *ne* value, using the -nh option.

#### Representing metadata with coloured DBGs

The subgraphs are enriched with metadata to make their interpretation easier. We use the node size to represent allele frequencies, *i.e.*, the proportion of genomes containing the unitig sequence. We describe the effect *β* of each unitig as estimated by the LMM using colours, in the spirit of the coloured DBGs [19]. Colours are continuously interpolated between red for unitigs with a strong positive effect and blue for those with a strong negative effect.

#### Annotating the subgraphs

DBGWAS can optionally integrate an automated annotation step using the Blast suite [60] (version 2.6.0+) on local user-defined protein (-pt-db option) or nucleic acid (-nt-db option) sequence databases. We annotate the subgraphs of interest by blasting each unitig sequence to the available databases. Users can then easily retrieve the annotations which are the most supported by the nodes in the subgraph, or with the lowest E-value. Importantly, DBGWAS works with any nucleotide or protein Fasta files as annotation databases straight away. However, users can customize the annotation databases by changing the Fasta sequences headers to make DBGWAS results more interpretable. A common example is compacting the annotation in the summary page by using abbreviations or gene class names, and expanding them to full names in the subgraph page. Other custom fields can also be included in the annotation table by adding specific tags to the headers. A detailed explanation on how to customize annotation databases for DBGWAS can be found in https://gitlab.com/leoisl/dbgwas/wikis/Customizing-annotation-databases. We also provide on the DBGWAS website a resistance determinant database built by merging the ResFinder, MEGARes, and ARG-ANNOT databases [61–63], and a subset of UniProt restricted to bacterial proteins [24]. Subgraphs discussed in the Results section were annotated using these databases.

#### Interactive visualisation

DBGWAS produces an interactive view of the enriched and annotated subgraphs, allowing the user to explore the graph topology together with information on each node: allele and phenotype frequencies, q-value, estimated effect, and annotation. The view is built using HTML, CSS, and several Javascript libraries, the main one being Cytoscape.js [64]. Results can be shared and visualised in a web browser. As a large number of components can be produced in one run of DBGWAS, we provide a summary page allowing users to preview and filter the subgraphs. Filtering can be based upon the minimum q-value of all unitigs in the component (min_*q*_), or based on the annotations. A complete description of the DBGWAS interactive interface is available in https://gitlab.com/leoisl/dbgwas/wikis/DBGWAS-web-based-interactive-visualization.

#### Re-running from *step 2* or *step 3*

It is possible to re-run a part of the analysis if a first run with the default values was unsatisfactory. The -skip1 option allows to re-run from the second step, for instance to compute the lineage effects (adding the -newick option). It is also possible to re-run only the third step by using the -skip2 option, for instance when the default *SFF* and *nh* values generated highly connected graphs, or if the annotation was incomplete.

### Datasets

We used in our experiments genome sequences from three bacterial species with various degrees of genome plasticity, from more clonal to more plastic: *M. tuberculosis*, *S. aureus*, and *P. aeruginosa*. We also built large datasets with random phenotypes for these 3 species, and used them only for time performance and memory usage assessment. All panels are summarised in Table 4.

**Table 4:** Microbial panels.

Species	Genome plasticity	Range of genome length	Panel name	Source	Phenotype	Number of available genomes
*M. tuberculosis*	very low	4.4 Mbp	TB	[35]	rifampicin	1,197
					isoniazid	1,287
					ethambutol	1,041
					streptomycin	1,166
					kanamycin	671
					ofloxacin	696
					ethionamide	420
					MDR	1,211
					XDR	689
			Large TB	[11]	random	5,000
*S. aureus*	low	2.7-3.1 Mbp	SA	[30]	methicillin	501
					ciprofloxacin	991
					erythromycin	991
					penicillin	991
					tetracycline	991
					fusidic acid	991
					trimethoprim	323
					gentamicin	991
					rifampin	991
					mupirocin	490
					vancomycin	501
			Large SA	[11]	random	9,000
*P. aeruginosa*	high	5.8-7.6 Mbp	PA	[65]	amikacin	280
					levofloxacin	117
					meropenem	280
					piperacillin	280
					colistin	164
					polymyxin B	117
					chloramphenicol	103
					cefepime	280
					fosfomycin	113
			Large PA	[11]	random	2,500

We selected 3 bacterial species with distinct levels of genome plasticity, and with antibiotic resistance phenotypes available for several drugs. For each species, we also created large datasets by computing random phenotypes for all available genome assemblies from NCBI RefSeq.

#### TB panel

*M. tuberculosis* (TB) is a human pathogen causing 1.7 million deaths each year [66]. This species is known for its apparent absence of horizontal gene transfer (HGT) and, accordingly, most of the reported resistance determinants are chromosomal mutations [67] in core genes or gene promoters. Intergenic regions are also described to be instrumental in multidrug-resistance (MDR) and extensively drug-resistant (XDR) phenotypes [9]. We use the PATRIC AMR phenotype data, as well as genome assemblies from their resource [35, 68]. We thus gather a total of 1302 genomes after filtering based on genome length. Phenotype data include isoniazid, rifampicin, streptomycin, ethambutol, ofloxacin, kanamycin and ethionamide resistance status. Except for the last three drugs, phenotype data are available for more than a thousand genomes. We reconstruct MDR and XDR phenotypes based on the WHO definition [66]. XDR phenotype could only be defined for 689/1302 strains as it required data for at least 4 drugs. Information on how phenotype data and genome assemblies were obtained is available on the PATRIC website.

#### SA panel

*S. aureus* is a human pathogen causing life-threatening infections. It is subject to HGT and many plasmids, mobile elements, and phage sequences have been described in its genome. However, this does not affect the species’ genome size, which is always close to 3 Mbp [69]. Most antibiotic resistance mechanisms are well determined by known variants, as shown in a previous study [30]. This study obtained an overall sensitivity of 97% for predicting 12 phenotypes from rules based on antibiotic marker mapping. We use this study panel of 992 strains obtained by merging their derivation and validation sets.

#### PA panel

*P. aeruginosa* is a ubiquitous bacterial species responsible for various types of infections. It is highly adaptable thanks to its ability to exchange genetic material within and between species [70]. The species accessory genome is particularly important both in terms of size and diversity, and carries more than half of the genetic determinants already described to confer resistance to antimicrobial drugs [7, 65, 71]. We use a panel of 282 strains, gathered from two collections which mostly include clinical strains: the bioMérieux collection [65] (n = 219) and the Pirnay collection [72] (n = 63). Genome assemblies and categorical phenotypes for 9 antibiotics are available [7]. Binarised phenotypes of amikacin resistance are available on the DBGWAS project page as an example for users.

#### Phenotype binarisation

Most available phenotypes are categorical, with S, I and R levels, respectively, for susceptible, intermediary, and resistant. We binarise them by assigning a zero value to susceptible strains (S) and one to others (I and R).

#### Large panels

We built large panels for the three species, in order to analyse the computational performance at a comprehensive scale. To do so, we gathered all genome assemblies of *M. tuberculosis* (5,504), *S. aureus* (9,331), and *P. aeruginosa* (2,802) available on the NCBI RefSeq bacterial genome repository [11], and removed poor quality genomes. For each panel, we generated random binary phenotypes. For a detailed time and memory assessment, we built several sub-panels from these three large panels at size points of 100, 250, 500, 1,000, 2,500, 5,000 and 9,000 genomes. To build these sub-panels, we sampled genomes uniformly from the panels. To take into account the variability among subsamplings, each sub-panel was randomly built 10 times.

### Resistome-based association studies

We benchmarked DBGWAS against a targeted approach to ensure its ability to retrieve all expected resistance determinants. We thus performed association studies under the same model, using as input a collection of known causal resistance SNPs and genes, defining the resistome.

In this validation study, we used bugwas with the same phenotypes and population structure matrix *W*, so the resistome-based analyses and DBGWAS only differ by their input variant matrix (unitigs versus SNPs or genes presence/absence).

For *P. aeruginosa* resistome, we use a variant matrix previously described [7], which includes presence/absence of known resistance gene variants, as well as the SNPs called against these reference gene variants. For *M. tuberculosis* resistome, we built the variant matrix using the same approach as for *P. aeruginosa* [7]: we called the SNPs from a list of 32 known resistance genes and promoters [34, 67, 73]. The time and memory usage required for the complete analysis (from the mapping of the resistance genes and positions on the genome assemblies to the association study) are provided in Tables 2 and 3.

We sort the annotated features by q-values. S6 and S7 Tables summarise all top variants using their q-value ranks, while Tables 2 and 3 report the annotations of all variants with a q-value < 0.05 for *P. aeruginosa* levofloxacin and *M. tuberculosis* streptomycin resistance, respectively.

### k-mer-based GWAS

#### pyseer

We installed pyseer [6, 36] commit ID d17602500a4530b0e68a679ed675fdb12942f56f (9 commits ahead of pyseer v1.1.1). pyseer pipeline is composed of four steps: 1) k-mer counting; 2) population structure estimation; 3) running pyseer; 4) downstream analysis. To use the correct parameters, we followed the pyseer tutorial (https://pyseer.readthedocs.io/en/master/tutorial.html). For k-mer counting, we used fsm-lite (https://github.com/nvalimak/fsm-lite), filtering out all k-mers with a minor allele frequency smaller than 1%. For population structure estimation, we used Mash v2.0 [74]. To run pyseer, we used 8 cores and a LRT p-value threshold of 0.05. Downstream analysis involved getting the k-mers which exceeded the significance threshold (which can be found using the scripts/count_patterns.py script), sorting them by LRT p-value, blasting them against the two databases presented in the Interpretation of significant unitigs (step 3) subsection, and keeping the best hit for each k-mer. For reproducibility purposes, the scripts we used to run pyseer can be found at https://gitlab.com/leoisl/DBGWAS_support/tree/master/scripts/pySEER.

#### HAWK

We firstly ran HAWK [13] v0.9.8-beta, as it allows correcting for population structure. Unfortunately, it was unable to find the known causal variants reported for *P. aeruginosa* levofloxacin and *M. tuberculosis* streptomycin resistances by other methods (see Tables 2 and 3). We therefore kept in our benchmarks an earlier version, HAWK v0.8.3-beta, which presented better qualitative performance for these two evaluated panels. HAWK pipeline is composed of five steps: 1) k-mer counting with a modified version of jellyfish [75]; 2) running HAWK; 3) assembling significant k-mers with ABYSS [76]; 4) getting statistics on the assembled sequences; 5) downstream analysis. The first four steps were performed as described in HAWK’s github page. However, in the first step, we had to remove the lower-count cutoff in jellyfish dump (parameter -L), since we are working with contigs and not reads. The last step was performed similarly as the one described for pyseer. For reproducibility purposes, the scripts we used to run HAWK v0.8.3-beta can be found at https://gitlab.com/leoisl/DBGWAS_support/tree/master/scripts/HAWK_0_8_3_beta.

## Supporting information

S1 Fig Alignment to a reference (when possible), cDBG, and k-mers obtained for similar (A) and very polymorphic genomes (B). In the first case, the 3 loci represented as polymorphic in the alignment lead to 3 bubble patterns in the cDBG, and numerous redundant k-mers. In the second case, genomes are so polymorphic that an alignment is not possible. The cDBG summarizes well the common regions and the links between them, while the collection of unique k-mers still contains redundancy.
(PDF)

S2 Fig Effect of *SFF* on the top subgraphs generated for *S. aureus* ciprofloxacin resistance. Annotation of the first subgraphs is strictly conserved (red for *parC*, green for *gyrA*, yellow for *norA* promoter region, blue for noncoding between *glmM* and *fmtB* and violet for transposase flanking regions).
(PDF)

S3 Fig Effect of *SFF* on the top subgraphs generated for *S. aureus* methicillin resistance. Only one subgraph, containing the *mecA* gene (highlighted in red) is generated for lower *SFF* values. Then several regions of the SCC*mec* cassette appear for *SFF* = 70, and are aggregated into a single subgraph for *SFF* ≥ 150. Green subgraphs do not concern the *mecA* MGE.
(PDF)

S4 Fig Effect of *SFF* on the top subgraphs generated for *S. aureus* penicillin resistance. Green subgraphs do not concern the *blaZ* MGE. Annotations are ordered by number of nodes carrying it. Yellow, orange and pink highlight *blaZ*, *blaR1* and *blaI*, respectively.
(PDF)

S5 Fig Effect of *SFF* on the top subgraphs generated for *S. aureus* erythromycin resistance. Only one subgraph, describing the *ermC* and its plasmid is outputted when *SFF* < 200. Green subgraphs do not concern the *ermC* MGE.
(PDF)

S6 Fig Effect of *SFF* on the top subgraphs generated for *P. aeruginosa* amikacin resistance. Nodes corresponding to *aac(6’)* gene are shown in a blue frame. When the *SFF* parameter increases, these nodes aggregate to others genes found at least once close to *aac(6’)*. The annotation of the following subgraphs are well conserved (same color legend as in S8 Fig).
(PDF)

S7 Fig Effect of *k* on the four first subgraphs obtained for TB rifampicin resistance. With a *k* value varying between 21 and 41, the first 3 subgraphs always have the same ordering, shape and annotation, as well as comparable q-values, although smaller q-values are observed for lower values of *k*. The number of significant unitigs per subgraph is also well conserved. The fourth top-rated subgraphs are not always the same: the *gyrA* mutation appears at a lower rank when *k* is smaller.
(PDF)

S8 Fig Effect of *k* on the five first subgraphs obtained for *P. aeruginosa* amikacin resistance. When *k* varies, the plasmid (yellow) and the mercury reductase and transposase (blue) remain among the five top-rated subgraphs. However, *k* has an effect on the aggregation of subgraphs corresponding to different genetic events: the mutation on *aac(6’)* gene (blue frame) always appears in the first subgraph but is merged with the large mercury reductase and transposase subgraph for *k* = 27, 39 and 41. The order of the subgraphs also varies with *k*: up to four ranks for some subgraphs, and others leave the top-5 list.
(PDF)

S9 Fig Large scale analysis on computational resources usage. This figure describes how DBGWAS scales in terms of time and memory usage for large datasets, containing up to 9,000 genomes. The large panels used here are described in the Large panels subsection of the Methods section. To understand better DBGWAS performance behaviour, we present performance curves for each panel at size points of 100, 250, 500, 1,000, 2,500, 5,000 and 9,000 genomes. The executions were done in a cluster, instead of a single machine, and used 8 cores each. In order to reduce subsampling and machine heterogeneity problems, each sub-panel was randomly built 10 times and we present the time and memory usage for all these executions. Although these two measures not only depends on the number of input genomes but also on their length and complexity, this figure allows estimations of the computational resources usage on small and large panels with different genome plasticities.
(PDF)

S1 Table DBGWAS time and maximal memory load on a single core. All runs presented in this table were executed with the default parameters, without optional steps (lineage effect analysis nor annotation of subgraphs), on a single *Intel(R) Xeon(R) CPU E5-2620 v3 @ 2.40GHz* core. The datasets are described in the Datasets subsection of the Methods section. DBGWAS ran in less than 2,5 hours for all experiments in our benchmark. The maximum memory load (given between parenthesis in the Runtime column) was 11 GB of RAM. The panel size and genome length (given between parenthesis in the Panel column) did not drive alone the running performances; the genome complexity played an important role as well. To view the gain in performance of DBGWAS when running on multiple (8) cores, see S2 Table.
(PDF)

S2 Table Benchmarking DBGWAS, pyseer and HAWK: Comparison of time and maximal memory load. The total execution time is presented with the maximal memory consumption in parenthesis, in order of GBs. For pyseer and HAWK, the time and memory for each step is also detailed. All tools were ran on a same machine with 8 *Intel(R) Xeon(R) CPU E5-2620 v3 @ 2.40GHz* cores, 315 GB of RAM and 1 TB of disk space. Each execution used all the 8 available cores. The datasets are described in the Datasets subsection of the Methods section. However, for the three large panels (Large TB, Large SA, and Large PA), here we just chose a random 2,500-genome sub-panel. Moreover, DBGWAS was ran with the default parameters, without optional steps (lineage effect analysis nor annotation of subgraphs). The parameters for pyseer and HAWK were the ones described in the k-mer-based GWAS subsection of the Methods section. We did not consider the time and memory consumed in the last step for these two tools (downstream analysis). The runs taking more than 5 days to finish were interrupted and are shown as *Timeout*. The runs that exceeded 1 TB of disk space were interrupted and are shown as *DQE* (Disk Quota Exceeded).
(PDF)

S3 Table DBGWAS results for *M. tuberculosis* resistance to antibiotics. For each antibiotic, top subgraphs were reported with their rank, the q-value of the unitig with the lowest q-value (min_*q*_), the corresponding estimated effect (estimated *β* of the linear model) and the number of susceptible (resp. resistant) strains harbouring this unitig (count per phenotype). The type of event represented by the subgraph, its annotation and some comments and references on this annotation were also provided. Comments were coloured if the annotation was previously described in antibiotic resistance literature: in green if this description concerned the tested antibiotic, in orange otherwise.
(XLS)

S4 Table DBGWAS results for *S. aureus* resistance to antibiotics. For each antibiotic, top subgraphs were reported with their rank, the q-value of the unitig with the lowest q-value (min_*q*_), the corresponding estimated effect (estimated *β* of the linear model) and the number of susceptible (resp. resistant) strains harbouring this unitig (count per phenotype). The type of event represented by the subgraph, its annotation and some comments and references on this annotation were also provided. Comments were coloured if the annotation was previously described in antibiotic resistance literature: in green if this description concerned the tested antibiotic, in orange otherwise.
(XLS)

S5 Table DBGWAS results for *P. aeruginosa* resistance to antibiotics. For each antibiotic, top subgraphs were reported with their rank, the q-value of the unitig with the lowest q-value (min_*q*_), the corresponding estimated effect (estimated *β* of the linear model) and the number of susceptible (resp. resistant) strains harbouring this unitig (count per phenotype). The type of event represented by the subgraph, its annotation and some comments and references on this annotation were also provided. Comments were coloured if the annotation was previously described in antibiotic resistance literature: in green if this description concerned the tested antibiotic, in orange otherwise.
(XLS)

S6 Table Resistome-based association study results for *M. tuberculosis* resistance to antibiotics. For each antibiotic, the 10 first features most associated to the phenotype were reported, with their rank, q-value, and estimated effect (estimated *β* of the linear model). The type of targeted variant, with its gene annotation were also provided. Comments were coloured if the annotation was previously described in antibiotic resistance literature: in green if this description concerned the tested antibiotic, in orange otherwise. The last column presents the corresponding subgraphs found by DBGWAS, with their rank and min_*q*_.
(XLS)

S7 Table Resistome-based association study results for *P. aeruginosa* resistance to antibiotics. For each antibiotic, the 10 first features most associated to the phenotype were reported, with their rank, q-value, and estimated effect (estimated *β* of the linear model). The type of targeted variant, with its gene annotation were also provided. Comments were coloured if the annotation was previously described in antibiotic resistance literature: in green if this description concerned the tested antibiotic, in orange otherwise. The last column presents the corresponding subgraphs found by DBGWAS, with their min_*q*_.
(XLS)

S8 Table Number of subgraphs generated using different significance thresholds. This table shows the number of subgraphs generated when defining the significant unitigs as the ones with the 100 lowest q-values (default *SFF* = 100, ‘top 100’) or when using a 5% false discovery rate (FDR) threshold (*SFF* = 0.05, ‘5% FDR’). Different datasets lead to different q-values, even by several orders of magnitude. For instance, a single FDR threshold leads to selecting a large number of unitigs generating several hundreds subgraphs for SA (*S. aureus*) panel.
(PDF)

S1 Appendix Evaluation of association models. (PDF)
