# Supplementary material for: A fast and agnostic method for bacterial genome-wide association studies: Bridging the gap between k-mers and genetic events
Source: PLoS Genet. 2018 Nov 12;14(11):e1007758. doi: 10.1371/journal.pgen.1007758 (PMC6258240; doi:10.1371/journal.pgen.1007758)
Supplement: S1 Fig — In the first case, the 3 loci represented as polymorphic in the alignment lead to 3 bubble patterns in the cDBG, and numerous redundant k-mers. In the second case, genomes are so polymorphic that an alignment is not possible. The cDBG summarizes well the common regions and the links between them, while the collection of unique k-mers still contains redundancy. (PDF) [file pgen.1007758.s001.pdf]

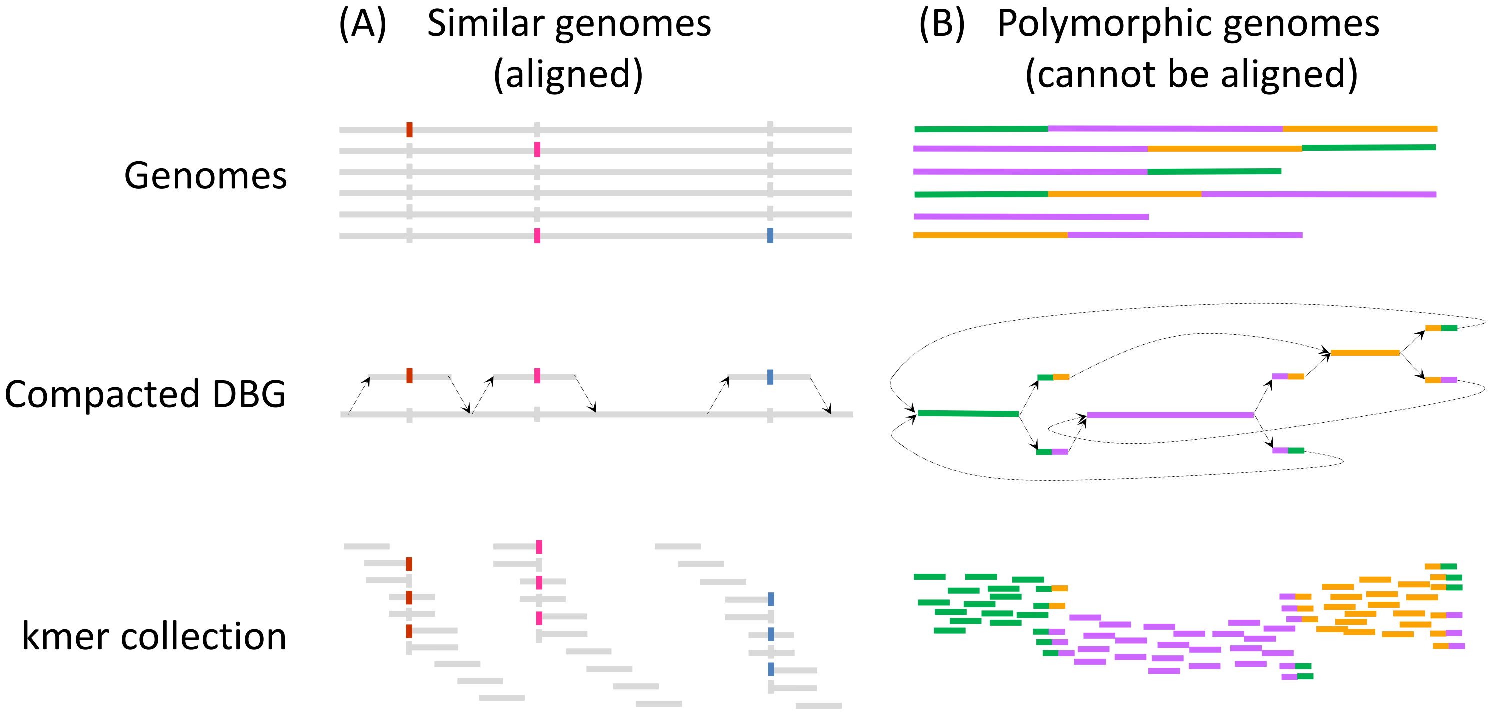
(A) Similar genomes (aligned)
(B) Polymorphic genomes (cannot be aligned)
Genomes
Compacted DBG
kmer collection
