# Supplementary material for: A fast and agnostic method for bacterial genome-wide association studies: Bridging the gap between k-mers and genetic events
Source: PLoS Genet. 2018 Nov 12;14(11):e1007758. doi: 10.1371/journal.pgen.1007758 (PMC6258240; doi:10.1371/journal.pgen.1007758)
Supplement: S2 Fig — Annotation of the first subgraphs is strictly conserved (red for parC, green for gyrA, yellow for norA promoter region, blue for noncoding between glmM and fmtB and violet for transposase flanking regions). (PDF) [file pgen.1007758.s002.pdf]

S. aureus ciprofloxacin

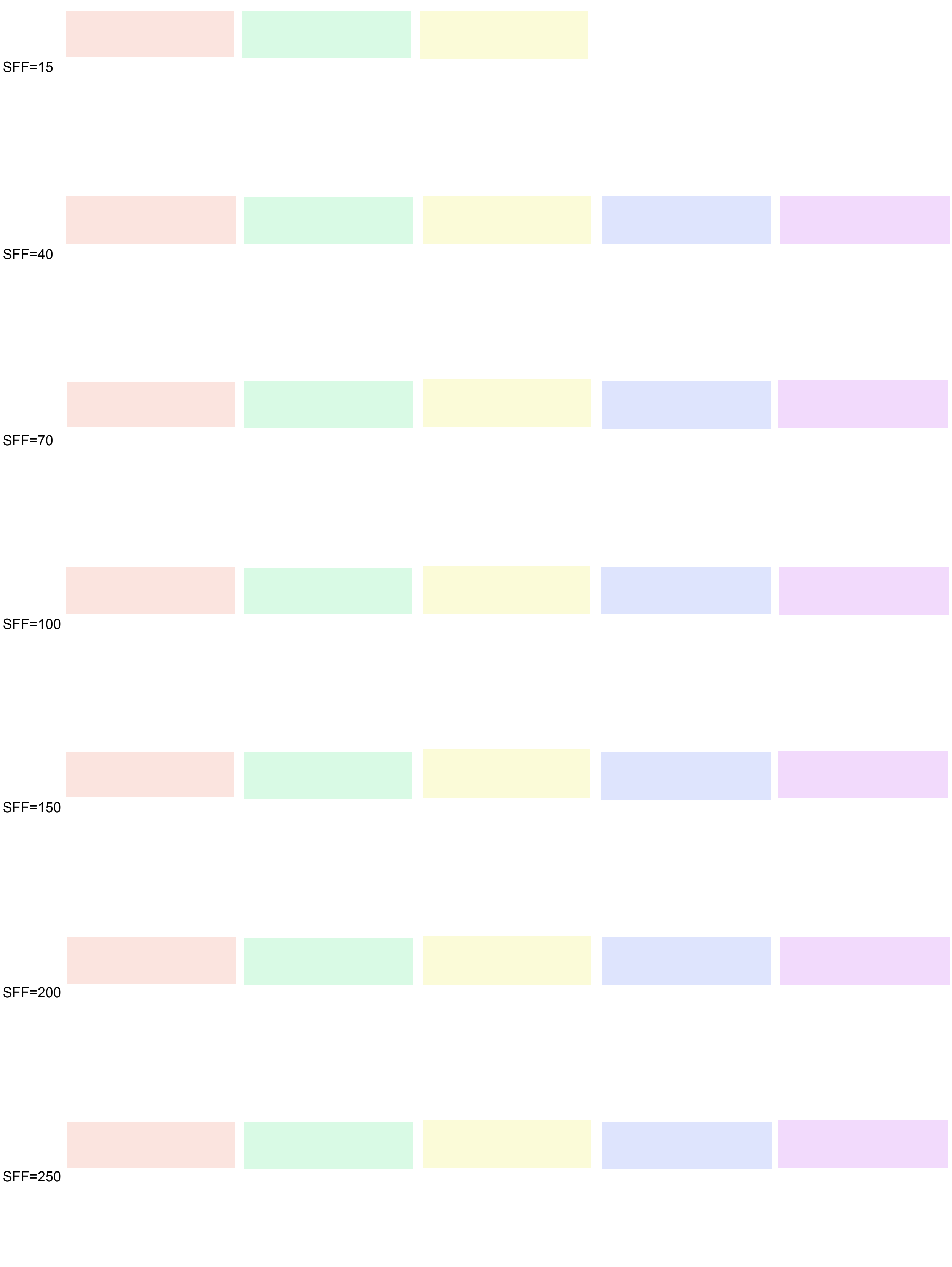

The raw DBGWAS results with the different values for SFF, which are summarised in this figure, are available at http://pbil.univ-lyon1.fr/datasets/DBGWAS_support/experiments/index.html#DBGWAS_all_results_different_SFF
