# Supplementary material for: A fast and agnostic method for bacterial genome-wide association studies: Bridging the gap between k-mers and genetic events
Source: PLoS Genet. 2018 Nov 12;14(11):e1007758. doi: 10.1371/journal.pgen.1007758 (PMC6258240; doi:10.1371/journal.pgen.1007758)
Supplement: S4 Fig — Green subgraphs do not concern the blaZ MGE. Annotations are ordered by number of nodes carrying it. Yellow, orange and pink highlight blaZ, blaR1 and blaI, respectively. (PDF) [file pgen.1007758.s004.pdf]

S. aureus penicillin

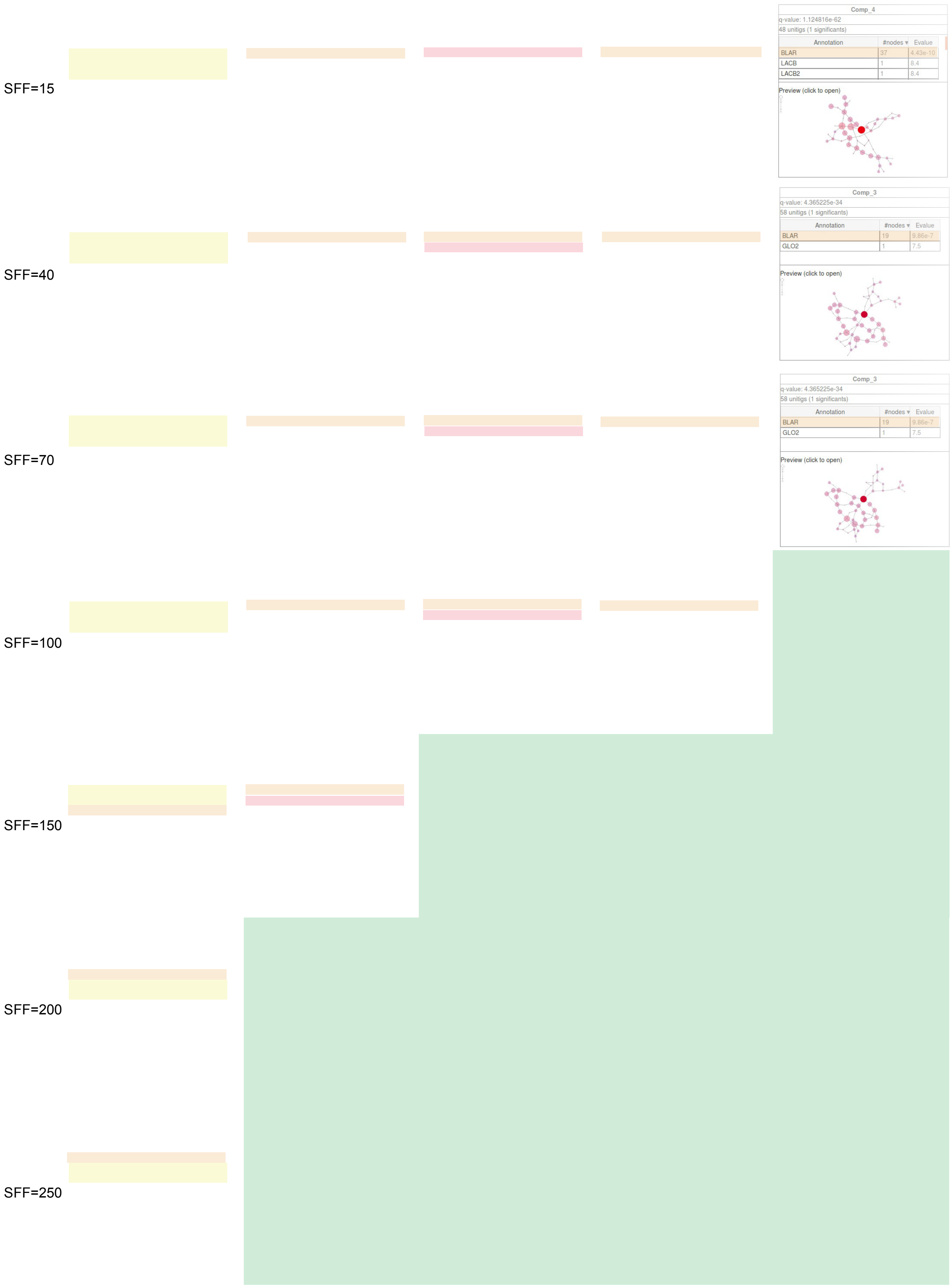

The raw DBGWAS results with the different values for SFF, which are summarised in this figure, are available at http://pbil.univ-lyon1.fr/datasets/DBGWAS_support/experiments/index.html#DBGWAS_all_results_different_SFF
