# Supplementary material for: A fast and agnostic method for bacterial genome-wide association studies: Bridging the gap between k-mers and genetic events
Source: PLoS Genet. 2018 Nov 12;14(11):e1007758. doi: 10.1371/journal.pgen.1007758 (PMC6258240; doi:10.1371/journal.pgen.1007758)
Supplement: S5 Fig — Only one subgraph, describing the ermC and its plasmid is outputted when SFF < 200. Green subgraphs do not concern the ermC MGE. (PDF) [file pgen.1007758.s005.pdf]

S. aureus erythromycin

SFF=15

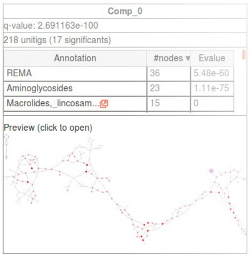

Comp_0
q-value: 2.691163e-100
218 unitigs (17 significants)

Annotation	#nodes	Evalue
REMA	36	5.48e-60
Aminoglycosides	23	1.11e-75
Macrolides,_lincosam...	15	0

Preview (click to open)

SFF=40

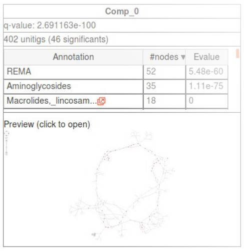

Comp_0
q-value: 2.691163e-100
402 unitigs (46 significants)

Annotation	#nodes	Evalue
REMA	52	5.48e-60
Aminoglycosides	35	1.11e-75
Macrolides,_lincosam...	18	0

Preview (click to open)

SFF=70

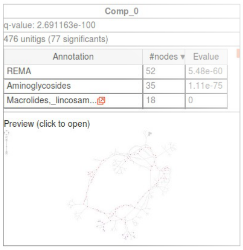

Comp_0
q-value: 2.691163e-100
476 unitigs (77 significants)

Annotation	#nodes	Evalue
REMA	52	5.48e-60
Aminoglycosides	35	1.11e-75
Macrolides,_lincosam...	18	0

Preview (click to open)

SFF=100

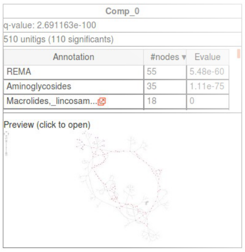

Comp_0
q-value: 2.691163e-100
510 unitigs (110 significants)

Annotation	#nodes	Evalue
REMA	55	5.48e-60
Aminoglycosides	35	1.11e-75
Macrolides,_lincosam...	18	0

Preview (click to open)

SFF=150

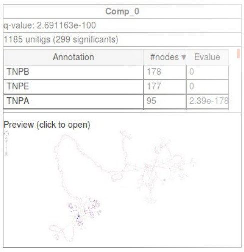

Comp_0
q-value: 2.691163e-100
1185 unitigs (299 significants)

Annotation	#nodes	Evalue
TNPB	178	0
TNPE	177	0
TNPA	95	2.39e-178

Preview (click to open)

SFF=200

SFF=250

The raw DBGWAS results with the different values for SFF, which are summarised in this figure, are available at http://pbil.univ-lyon1.fr/datasets/DBGWAS_support/experiments/index.html#DBGWAS_all_results_different_SFF
