# Supplementary material for: A fast and agnostic method for bacterial genome-wide association studies: Bridging the gap between k-mers and genetic events
Source: PLoS Genet. 2018 Nov 12;14(11):e1007758. doi: 10.1371/journal.pgen.1007758 (PMC6258240; doi:10.1371/journal.pgen.1007758)
Supplement: S6 Fig — Nodes corresponding to aac(6’) gene are shown in a blue frame. When the SFF parameter increases, these nodes aggregate to others genes found at least once close to aac(6’). The annotation of the following subgraphs are well conserved (same color legend as in S8 Fig). (PDF) [file pgen.1007758.s006.pdf]

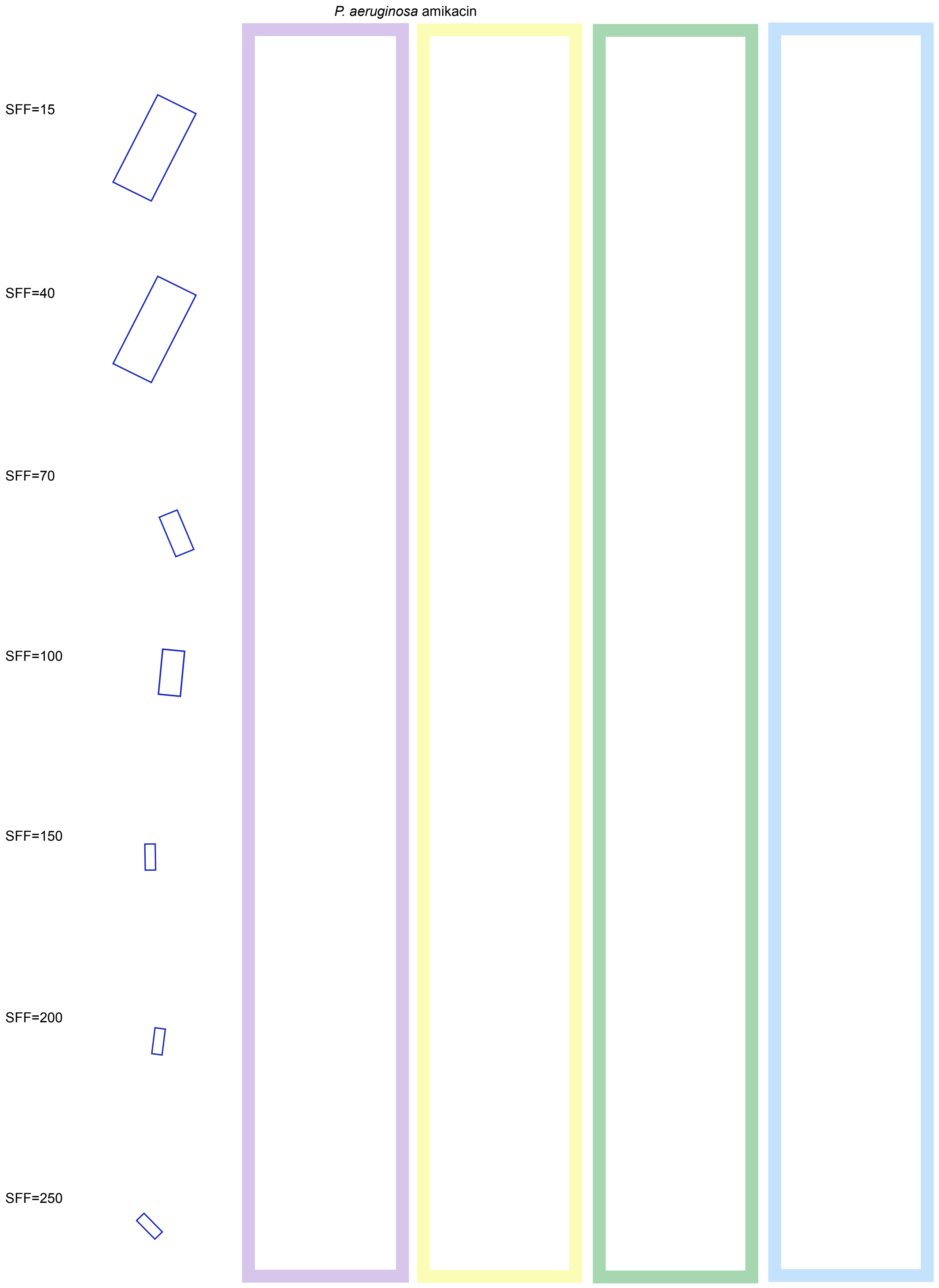

The raw DBGWAS results with the different values for SFF, which are summarised in this figure, are available at http://pbil.univ-lyon1.fr/datasets/DBGWAS_support/experiments/index.html#DBGWAS_all_results_different_SFF
