# Supplementary material for: A fast and agnostic method for bacterial genome-wide association studies: Bridging the gap between k-mers and genetic events
Source: PLoS Genet. 2018 Nov 12;14(11):e1007758. doi: 10.1371/journal.pgen.1007758 (PMC6258240; doi:10.1371/journal.pgen.1007758)
Supplement: S7 Fig — With a k value varying between 21 and 41, the first 3 subgraphs always have the same ordering, shape and annotation, as well as comparable q-values, although smaller q-values are observed for lower values of k. The number of significant unitigs per subgraph is also well conserved. The fourth top-rated subgraphs are not always the same: the gyrA mutation appears at a lower rank when k is smaller. (PDF) [file pgen.1007758.s007.pdf]

k=21	**Comp_3**<br>q-value: 1.922401e-75<br>Annotations on significant nodes:<br>RPOB<br>RPOB1<br>Preview (click to open)<br>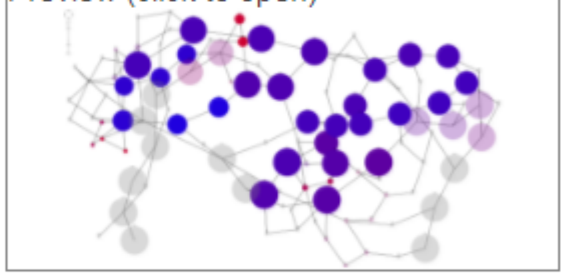	**Comp_17**<br>q-value: 4.201452e-21<br>Annotations on significant nodes:<br>KATG<br>KATG1<br>Preview (click to open)<br>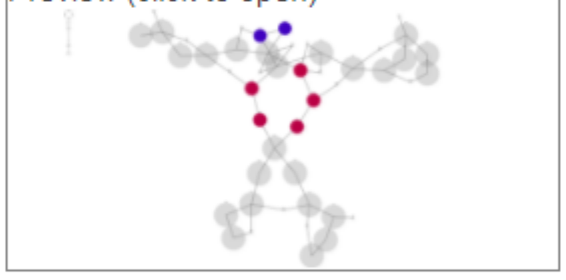	**Comp_27**<br>q-value: 1.205113e-09<br>Annotations on significant nodes:<br>EMBB<br>EMBC<br>Preview (click to open)<br>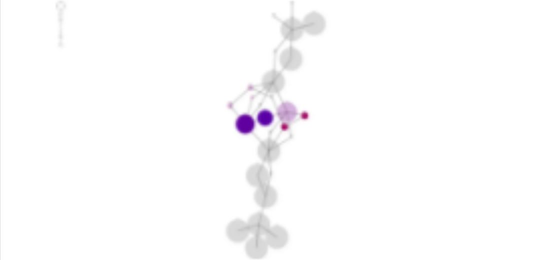	**Comp_25**<br>q-value: 6.470231e-02<br>Annotations on significant nodes: **No annotations found.**<br>Preview (click to open)<br>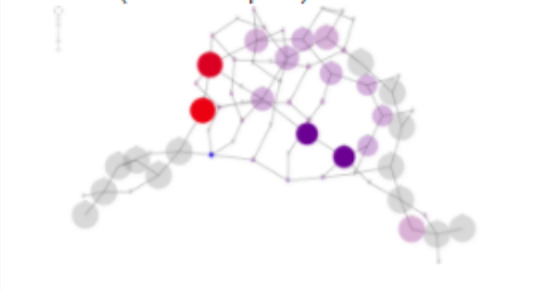
k=23	**Comp_5**<br>q-value: 1.026734e-74<br>Annotations on significant nodes:<br>RPOB<br>RPOB1<br>Preview (click to open)<br>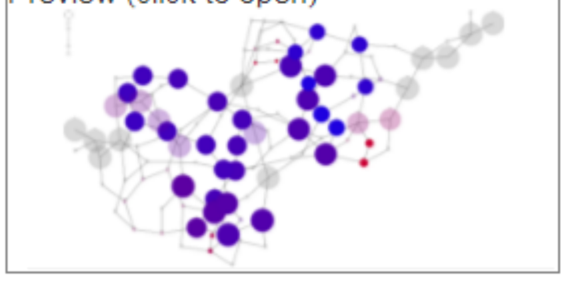	**Comp_22**<br>q-value: 1.056213e-21<br>Annotations on significant nodes:<br>KATG<br>KATG1<br>Preview (click to open)<br>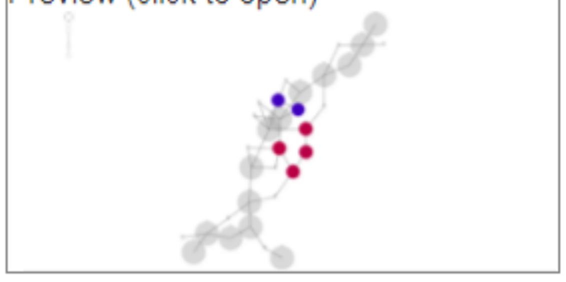	**Comp_10**<br>q-value: 2.370850e-09<br>Annotations on significant nodes:<br>EMBB<br>EMBC<br>Preview (click to open)<br>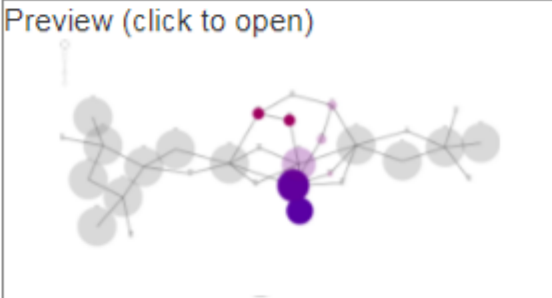	**Comp_21**<br>q-value: 3.841875e-02<br>Annotations on significant nodes: **No annotations found.**<br>Preview (click to open)<br>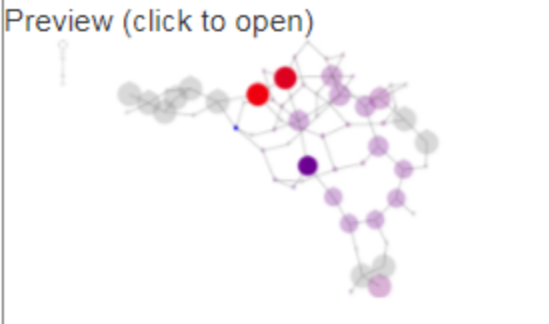
k=27	**Comp_6**<br>q-value: 1.447705e-71<br>Annotations on significant nodes:<br>RPOB<br>RPOB1<br>Preview (click to open)<br>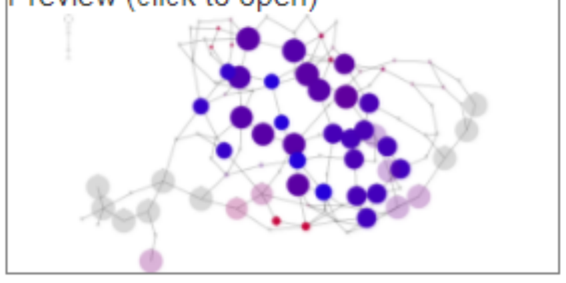	**Comp_8**<br>q-value: 5.003823e-22<br>Annotations on significant nodes:<br>KATG<br>KATG1<br>Preview (click to open)<br>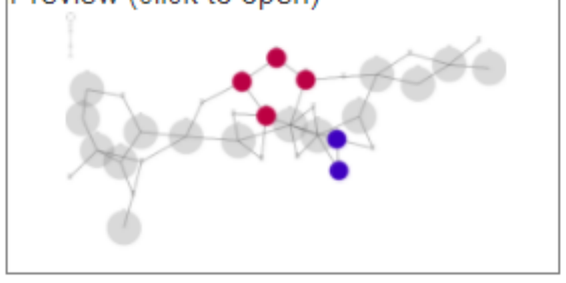	**Comp_21**<br>q-value: 1.912084e-09<br>Annotations on significant nodes:<br>EMBA<br>EMBB<br>Preview (click to open)<br>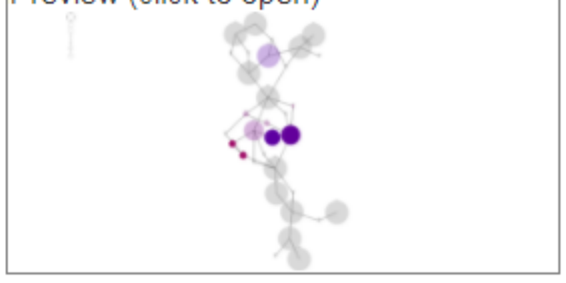	**Comp_14**<br>q-value: 3.713325e-02<br>Annotations on significant nodes:<br>Fluoroquinolones<br>GYRA<br>Preview (click to open)<br>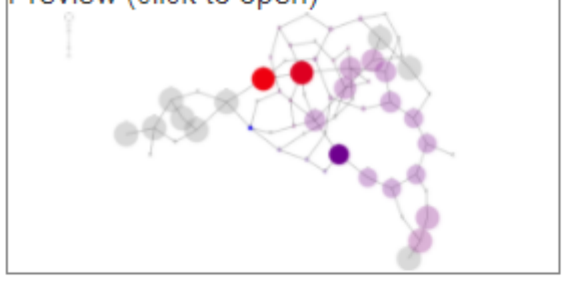
k=31	**Comp_2**<br>q-value: 4.837907e-70<br>Annotations on significant nodes:<br>RPOB<br>RPOB1<br>Preview (click to open)<br>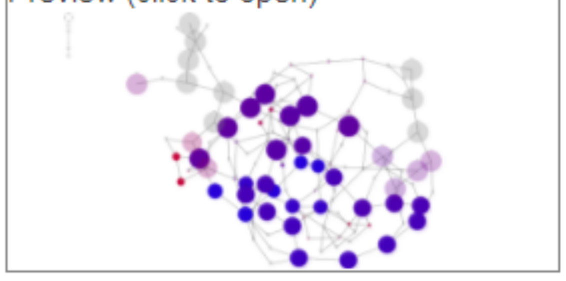	**Comp_17**<br>q-value: 4.346090e-20<br>Annotations on significant nodes:<br>KATG<br>KATG1<br>Preview (click to open)<br>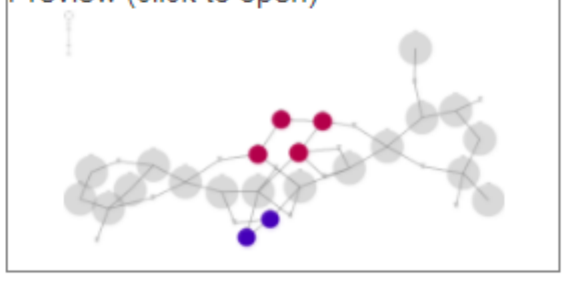	**Comp_14**<br>q-value: 4.023152e-08<br>Annotations on significant nodes:<br>EMBA<br>EMBB<br>Preview (click to open)<br>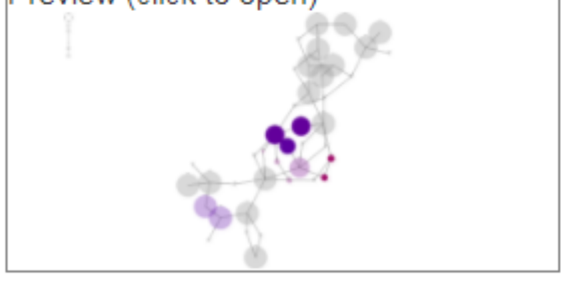	**Comp_19**<br>q-value: 1.790651e-02<br>Annotations on significant nodes: **No annotations found.**<br>Preview (click to open)<br>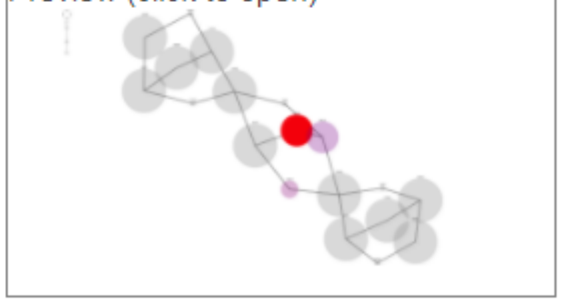
k=35	**Comp_3**<br>q-value: 3.314729e-66<br>Annotations on significant nodes:<br>RPOB<br>RPOB1<br>Preview (click to open)<br>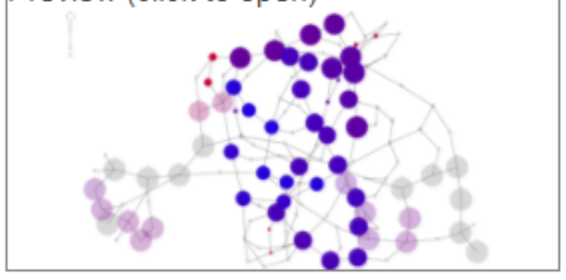	**Comp_19**<br>q-value: 6.722259e-19<br>Annotations on significant nodes:<br>KATG<br>KATG1<br>Preview (click to open)<br>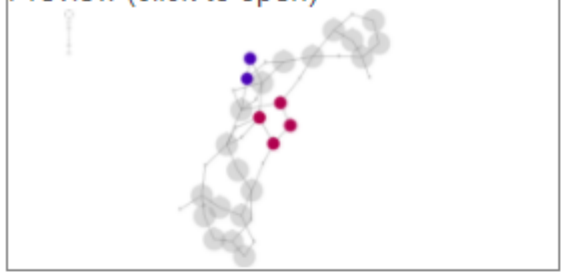	**Comp_4**<br>q-value: 2.857538e-07<br>Annotations on significant nodes:<br>EMBA<br>EMBB<br>Preview (click to open)<br>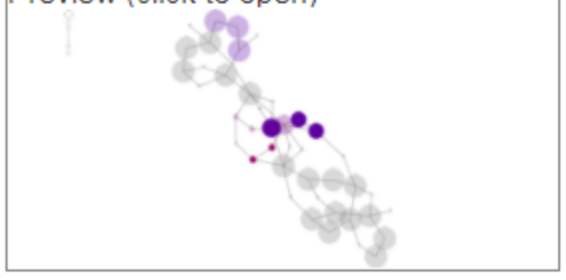	**Comp_15**<br>q-value: 1.309185e-02<br>Annotations on significant nodes: **No annotations found.**<br>Preview (click to open)<br>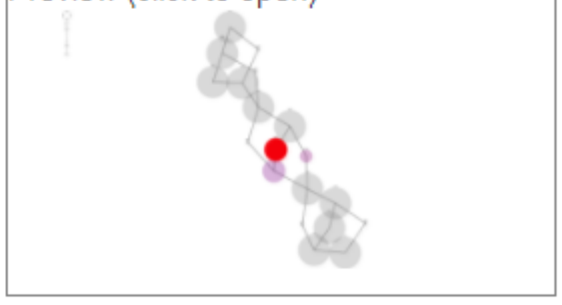
k=39	**Comp_8**<br>q-value: 4.570378e-64<br>Annotations on significant nodes:<br>RPOB<br>RPOB1<br>Preview (click to open)<br>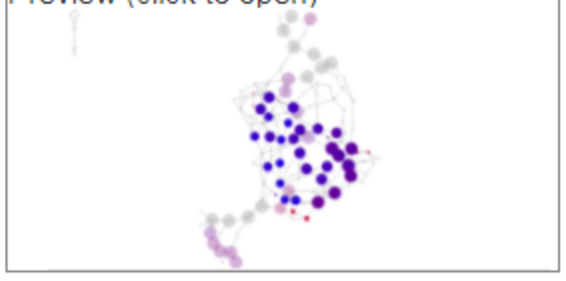	**Comp_10**<br>q-value: 3.697165e-19<br>Annotations on significant nodes:<br>KATG<br>KATG1<br>Preview (click to open)<br>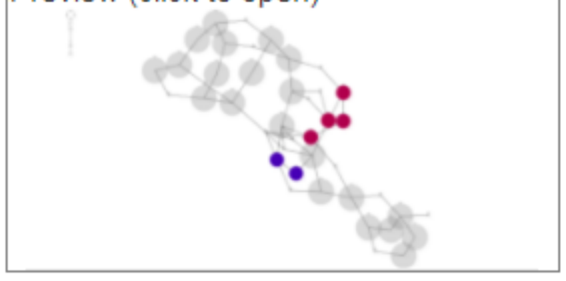	**Comp_20**<br>q-value: 1.195064e-07<br>Annotations on significant nodes:<br>EMBA<br>EMBB<br>Preview (click to open)<br>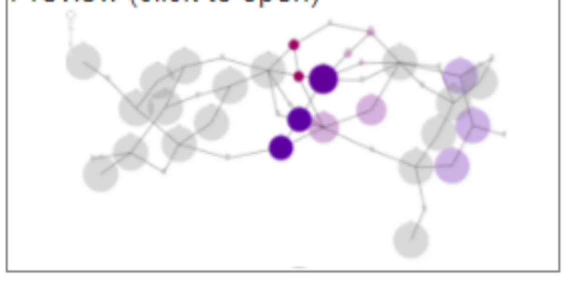	**Comp_1**<br>q-value: 1.834660e-02<br>Annotations on significant nodes:<br>SYM<br>Preview (click to open)<br>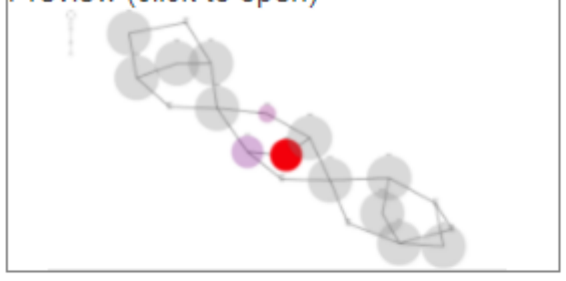
k=41	**Comp_2**<br>q-value: 5.831837e-60<br>Annotations on significant nodes:<br>RPOB<br>RPOB1<br>Preview (click to open)<br>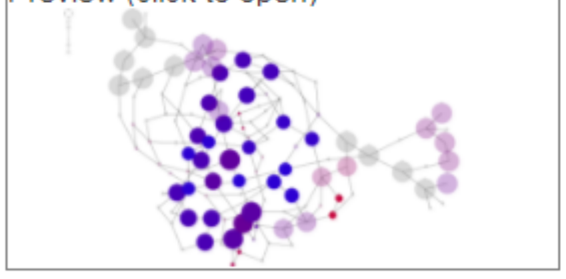	**Comp_15**<br>q-value: 3.719410e-18<br>Annotations on significant nodes:<br>KATG<br>KATG1<br>Preview (click to open)<br>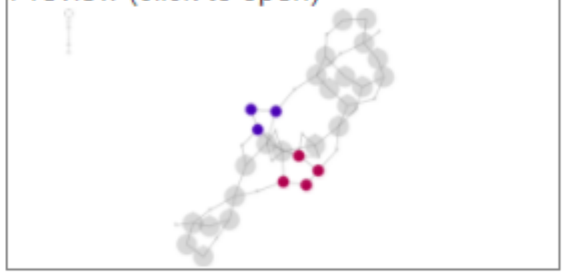	**Comp_4**<br>q-value: 1.682796e-08<br>Annotations on significant nodes:<br>EMBA<br>EMBB<br>Preview (click to open)<br>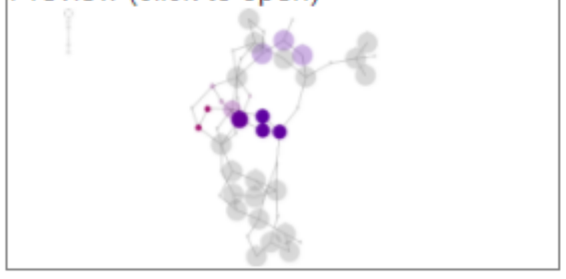	**Comp_20**<br>q-value: 1.726185e-02<br>Annotations on significant nodes:<br>SYM<br>Preview (click to open)<br>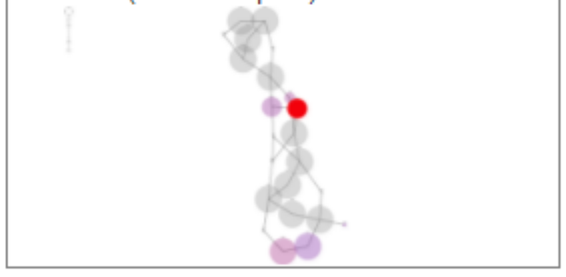
