# Supplementary figures and images for: A fast and agnostic method for bacterial genome-wide association studies: Bridging the gap between k-mers and genetic events
Source: PLoS Genet. 2018 Nov 12;14(11):e1007758. doi: 10.1371/journal.pgen.1007758 (PMC6258240; doi:10.1371/journal.pgen.1007758)

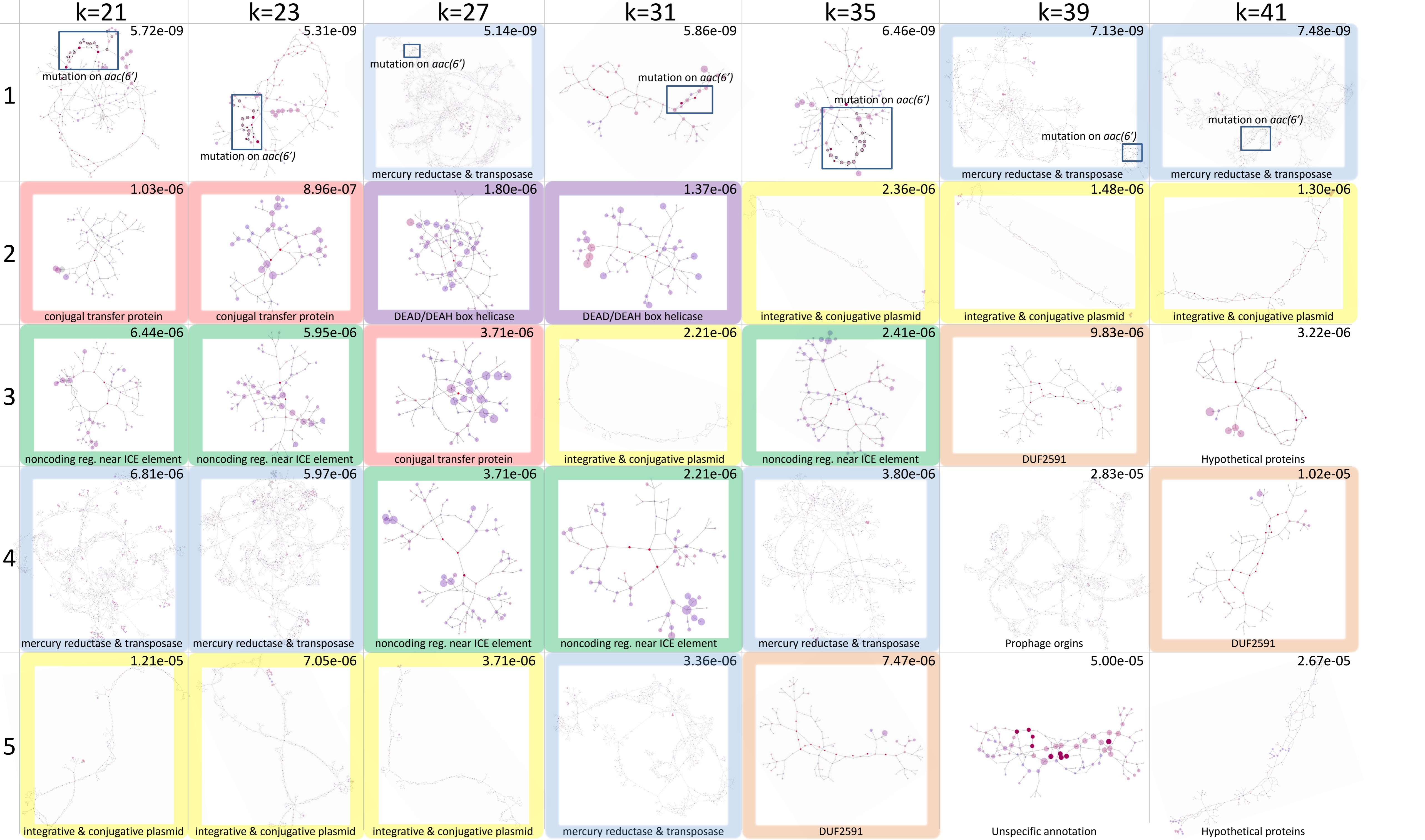

Supplement: S8 Fig — When k varies, the plasmid (yellow) and the mercury reductase and transposase (blue) remain among the five top-rated subgraphs. However, k has an effect on the aggregation of subgraphs corresponding to different genetic events: the mutation on aac(6’) gene (blue frame) always appears in the first subgraph but is merged with the large mercury reductase and transposase subgraph for k = 27, 39 and 41. The order of the subgraphs also varies with k: up to four ranks for some subgraphs, and others leave the top-5 list. (PDF) [file pgen.1007758.s008.pdf]

Computing Time

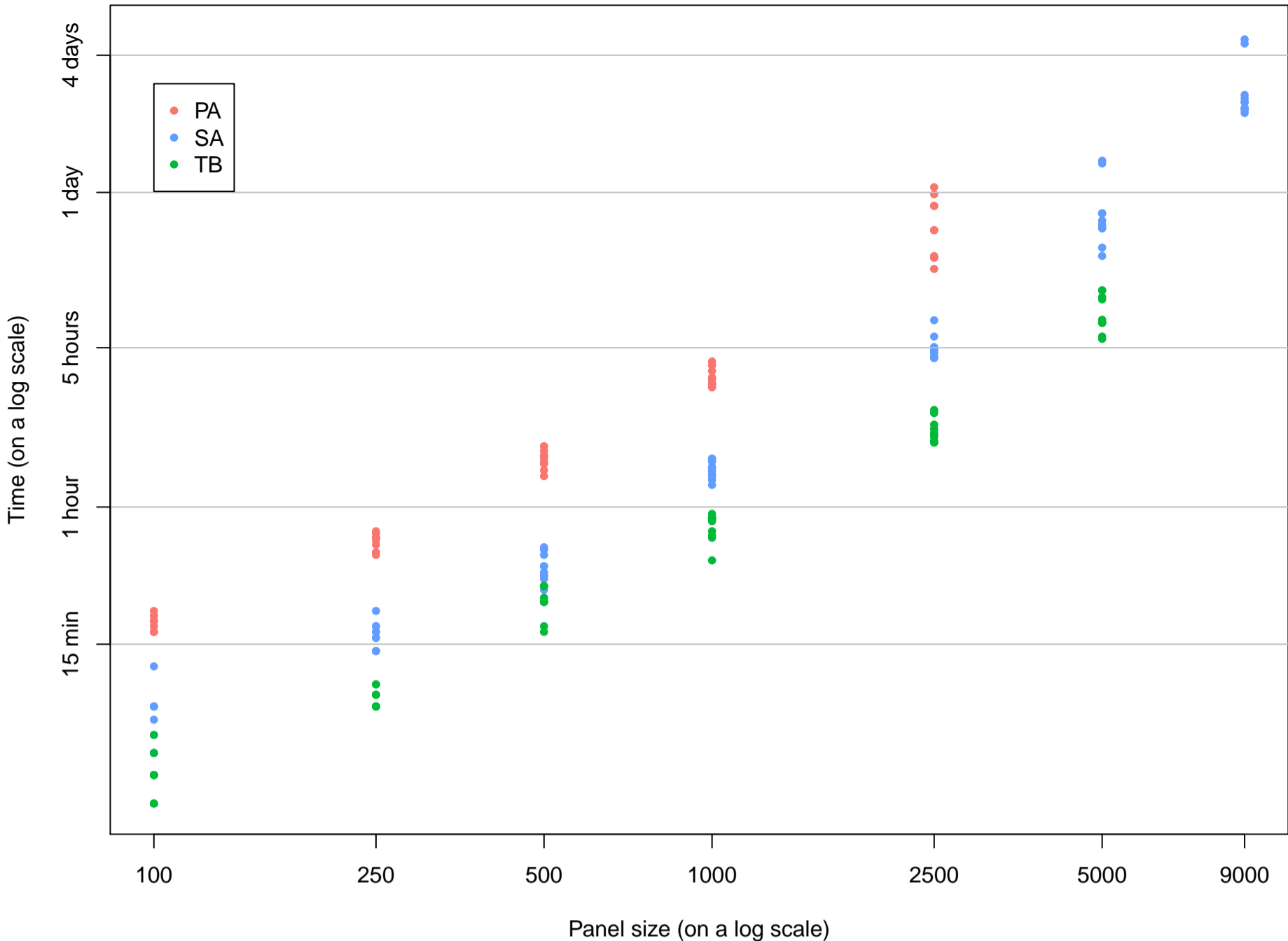

(A)

Memory usage

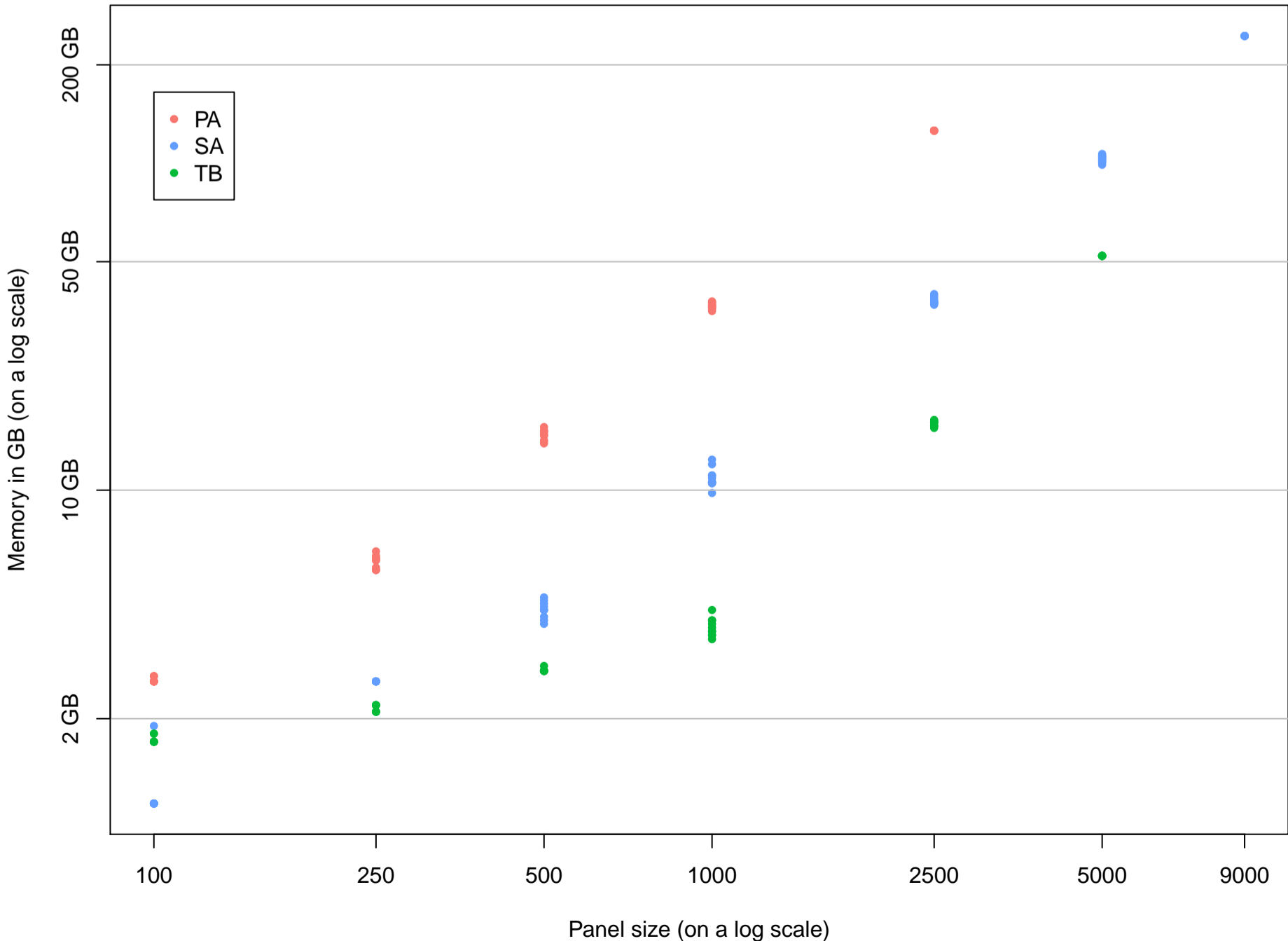

(B)

Supplement: S9 Fig — This figure describes how DBGWAS scales in terms of time and memory usage for large datasets, containing up to 9,000 genomes. The large panels used here are described in the Large panels subsection of the Methods section. To understand better DBGWAS performance behaviour, we present performance curves for each panel at size points of 100, 250, 500, 1,000, 2,500, 5,000 and 9,000 genomes. The executions were done in a cluster, instead of a single machine, and used 8 cores each. In order to reduce subsampling and machine heterogeneity problems, each sub-panel was randomly built 10 times and we present the time and memory usage for all these executions. Although these two measures not only depends on the number of input genomes but also on their length and complexity, this figure allows estimations of the computational resources usage on small and large panels with different genome plasticities. (PDF) [file pgen.1007758.s009.pdf]
