# Supplementary material for: A fast and agnostic method for bacterial genome-wide association studies: Bridging the gap between k-mers and genetic events
Source: PLoS Genet. 2018 Nov 12;14(11):e1007758. doi: 10.1371/journal.pgen.1007758 (PMC6258240; doi:10.1371/journal.pgen.1007758)
Supplement: S1 Table — All runs presented in this table were executed with the default parameters, without optional steps (lineage effect analysis nor annotation of subgraphs), on a single Intel(R) Xeon(R) CPU E5-2620 v3 @ 2.40GHz core. The datasets are described in the Datasets subsection of the Methods section. DBGWAS ran in less than 2,5 hours for all experiments in our benchmark. The maximum memory load (given between parenthesis in the Runtime column) was 11 GB of RAM. The panel size and genome length (given between parenthesis in the Panel column) did not drive alone the running performances; the genome complexity played an important role as well. To view the gain in performance of DBGWAS when running on multiple (8) cores, see S2 Table. (PDF) [file pgen.1007758.s010.pdf]

Panel (gen len Mbp)	Phenotype	Panel size	k-mers in M	Unitigs in M	Patterns[1] in M	Runtime on 1 core (mem GB)
TB (4.4)	ethambutol	1041	8.22	0.36	0.05	1h28m (3.5)
	streptomycin	1166	8.32	0.37	0.05	1h40m (4)
	rifampicin	1197	8.29	0.37	0.05	1h42m (4.1)
	ethionamide	420	5.74	0.13	0.02	32m (3.4)
SA (2.7-3.1)	ciprofloxacin	991	23.13	1.12	0.48	2h15m (11)
	erythromycin	991	23.13	1.12	0.48	2h10m (11)
	methicillin	501	17.85	0.81	0.30	51m (4.1)
PA (5.8-7.6)	meropenem	280	54.46	2.36	1.01	1h37m (7.6)
	levofloxacin	117	41.40	1.70	0.64	42m (2.9)
	amikacin	280	54.46	2.36	1.01	1h37m (7.6)

[1]Patterns are the columns of the X matrix, containing the unique unitig minor allele description profiles (see *Methods* section). Association tests are computed on the patterns.
