# Supplementary material for: A fast and agnostic method for bacterial genome-wide association studies: Bridging the gap between k-mers and genetic events
Source: PLoS Genet. 2018 Nov 12;14(11):e1007758. doi: 10.1371/journal.pgen.1007758 (PMC6258240; doi:10.1371/journal.pgen.1007758)
Supplement: S2 Table — The total execution time is presented with the maximal memory consumption in parenthesis, in order of GBs. For pyseer and HAWK, the time and memory for each step is also detailed. All tools were ran on a same machine with 8 Intel(R) Xeon(R) CPU E5-2620 v3 @ 2.40GHz cores, 315 GB of RAM and 1 TB of disk space. Each execution used all the 8 available cores. The datasets are described in the Datasets subsection of the Methods section. However, for the three large panels (Large TB, Large SA, and Large PA), here we just chose a random 2,500-genome sub-panel. Moreover, DBGWAS was ran with the default parameters, without optional steps (lineage effect analysis nor annotation of subgraphs). The parameters for pyseer and HAWK were the ones described in the k-mer-based GWAS subsection of the Methods section. We did not consider the time and memory consumed in the last step for these two tools (downstream analysis). The runs taking more than 5 days to finish were interrupted and are shown as Timeout. The runs that exceeded 1 TB of disk space were interrupted and are shown as DQE (Disk Quota Exceeded). (PDF) [file pgen.1007758.s011.pdf]

Panel	Phenotype	**DBGWAS**	pyseer				HAWK				
			fsm-lite	Mash v2.0	pyseer	**Total**	Count k-mers	HAWK	ABYSS	Stats	**Total**
TB	ethambutol	**37m (3.8)**	6h48m (93.6)	4m (0.2)	6h10m (3.6)	**13h02m (93.6)**	2h11m (1.5)	26m (3.7)	1m (0.3)	5m (0.1)	**2h43m (3.7)**
	streptomycin	**42m (4.3)**	7h47m (102.4)	5m (0.2)	6h22m (4.1)	**14h14m (102.4)**	2h26m (1.5)	25m (3.7)	1m (0.3)	9m (0.1)	**3h01m (3.7)**
	rifampicin	**43m (4.4)**	8h01m (104.7)	5m (0.2)	7h45m (4.1)	**15h51m (104.7)**	2h30m (1.5)	25m (3.7)	1m (0.3)	14m (0.1)	**3h10m (3.7)**
	ethionamide	**12m (3.5)**	2h18m (34.8)	1m (0.1)	1h35m (1.3)	**3h54m (34.8)**	53m (1.5)	17m (3.5)	1m (0.3)	1m (0.1)	**1h12m (3.5)**
Large TB	random	**1h44m (15.7)**	23h31m (215.8)	14m (0.9)	6h13m (11.3)	**29h58m (215.8)**	5h15m (1.5)	48m (4.2)	1m (0.3)	1m (0.1)	**6h05m (4.2)**
SA	ciprofloxacin	**1h16m (11.2)**	6h31m (53.8)	3m (0.2)	Timeout	**Timeout**	1h31m (1.5)	27m (4.0)	1m (0.4)	22h11m (0.1)	**24h10m (4.0)**
	erythromycin	**1h17m (11.2)**	6h32m (53.8)	3m (0.2)	Timeout	**Timeout**	1h31m (1.5)	26m (4.1)	1m (0.3)	7h56m (0.1)	**9h54m (4.1)**
	methicillin	**29m (4.3)**	3h15m (27.1)	1m (0.1)	Timeout	**Timeout**	46m (1.5)	19m (3.8)	1m (0.3)	8h29m (0.1)	**9h35m (3.8)**
Large SA	random	**5h11m (37.4)**	18h35m (149.5)	12m (0.9)	26h53m (6.8)	**45h40m (149.5)**	3h51m (1.5)	41m (5.8)	1m (0.3)	1m (0.1)	**4h34m (5.8)**
PA	meropenem	**49m (8.0)**	6h32m (34.7)	2m (0.1)	33h59m (0.5)	**40h33m (34.7)**	50m (1.5)	21m (4.5)	1m (0.3)	1m (0.1)	**1h13m (4.5)**
	levofloxacin	**21m (3.2)**	3h03m (14.5)	1m (0.1)	21h18m (0.3)	**24h22m (14.5)**	21m (1.5)	16m (4.2)	1m (0.3)	1m (0.1)	**39m (4.2)**
	amikacin	**51m (8.0)**	5h59m (34.7)	2m (0.1)	46h44m (0.5)	**52h45m (34.7)**	50m (1.5)	22m (4.5)	1m (0.3)	1m (0.1)	**1h14m (4.5)**
Large PA	random	**13h21m (125.8)**	DQE	DQE	DQE	**DQE**	7h24m (1.5)	2h05m (8.6)	1m (0.3)	1m (0.1)	**9h31m (8.6)**
