# Supplementary material for: A fast and agnostic method for bacterial genome-wide association studies: Bridging the gap between k-mers and genetic events
Source: PLoS Genet. 2018 Nov 12;14(11):e1007758. doi: 10.1371/journal.pgen.1007758 (PMC6258240; doi:10.1371/journal.pgen.1007758)
Supplement: S8 Table — This table shows the number of subgraphs generated when defining the significant unitigs as the ones with the 100 lowest q-values (default SFF = 100, ‘top 100’) or when using a 5% false discovery rate (FDR) threshold (SFF = 0.05, ‘5% FDR’). Different datasets lead to different q-values, even by several orders of magnitude. For instance, a single FDR threshold leads to selecting a large number of unitigs generating several hundreds subgraphs for SA (S. aureus) panel. (PDF) [file pgen.1007758.s017.pdf]

Panel	Phenotype	number of subgraphs	
		top 100	5% FDR
TB	MDR	28	3
	XDR	26	23
	ethambutol	23	23
	streptomycin	17	24
	rifampicin	25	6
SA	ciprofloxacin	163	1877
	erythromycin	1	444
	methicillin	4	352
PA	meropenem	70	1
	levofloxacin	56	5
	amikacin	59	397
