# Supplementary material for: A fast and agnostic method for bacterial genome-wide association studies: Bridging the gap between k-mers and genetic events
Source: PLoS Genet. 2018 Nov 12;14(11):e1007758. doi: 10.1371/journal.pgen.1007758 (PMC6258240; doi:10.1371/journal.pgen.1007758)
Supplement: S1 Appendix — (PDF) [file pgen.1007758.s018.pdf]

S1 Appendix. Evaluation of association models

We evaluate two models controlling for population structure: (i) using the first principal components as covariates with a fixed effect [1] (implemented in the PLINK software [2]) and (ii) linear mixed models [3–5], and compare them to (iii) a model not accounting for the population structure.

We evaluate the three models by their ability to detect (a) true positive unitigs simulated under different population structures, and (b) unitigs from real data mapping genuine variants described in the literature.

Evaluated models

Let $\mathbf{Z}$ be the full matrix of unitig minor allele frequency patterns (before de-duplication) and $\mathbf{X}$ be the matrix of unique patterns (after de-duplication) as defined in the *Methods* section of the main manuscript. For each pattern X_{ij}, we test $H_0 : \beta = 0$ versus $H_1 : \beta \neq 0$ in the following linear model, relating binarized antibiotic susceptibility phenotypes to X_{ij} candidate genetic determinant and population structure:

$$Y_i = X_{ij}\beta + W_i^T\alpha + \varepsilon_{ij}, \quad j = 1, \ldots, p \tag{1}$$

with $\varepsilon_{ij} \overset{\text{iid}}{\sim} \mathcal{N}(0, \sigma^2)$, $\sigma^2 > 0$, β the effect of the tested candidate on the phenotype, $W \in \mathbb{R}^l$ a factor representing the population structure, and $\alpha \in \mathbb{R}^l$ the effect of this population structure on the phenotype.

Denoting $\mathbf{Z} = USV^\top$ the singular value decomposition (SVD) of $\mathbf{Z}$, we use:

(i) $W = U_q$ (matrix formed by the first q columns of U) and a fixed effect α;

(ii) $W = US$ and a random effect $\alpha \sim \mathcal{N}(0, \sigma_a^2)$, $\sigma_a^2 > 0$;

(iii) $\alpha = 0$.

For the first two models, we compute p-values for H_0 using a likelihood ratio test. For the random effect model, we use bugwas [5] to test H_0, providing the pre-computed population structure W as described above.

Simulated data

We simulate resistance phenotypes based on the 282 *P. aeruginosa* genomes, arbitrarily fixing which patterns X have a non-zero effect β on the phenotype Y: we sample the phenotype Y_i of each sample i from a multivariate logistic model:

$$Y_i \sim \mathcal{B}(\pi_i), \quad \pi_i = \frac{1}{1 + e^{-\mathbf{X}_\mathbf{i}\beta - W_i\alpha}}. \tag{2}$$

We generate synthetic data under model (2) with two scenarios. The first scenario illustrates a case where there is a population effect on the observed resistance, which is not explained by the set of patterns in the tested design X. The second scenario illustrates the case where there is little population effect observed on the phenotype, except for that caused by the association of modeled causal patterns X with W, *i.e.*, outside of $\mathbf{X}\beta$ in (2).

To simulate the first scenario, we arbitrarily assign the 2nd and 6th columns of $W = U\Lambda^{\frac{1}{2}}$ to have non-zero effects α. We then select 10 distinct patterns from $\mathbf{X}$ as true determinants. To do so, we compute the largest dot product of each pattern with the first six columns of W, and choose our true determinants among those whose largest dot product is below the fifth percentile of dot products calculated across all patterns.

This allows us to simulate the case where true determinants are independent from the population structure (their effect is not inflated by the $W\alpha$ term). The odd ratios e^{β_j} are fixed to 6 for these patterns. We also randomly select 290 patterns from $\mathbf{X}$ as non-determinants, *i.e.*, with a $\beta_j = 0$ effect in the model, so $p = 300$ in our simulations. The population structure can lead to spurious discoveries, as we do not control the dot product between columns of W and these patterns with zero effect. Finally in order to control the amplitude of the population effect, we normalize $W\alpha$ to 6 times the median value of the $|\mathbf{X^j}\beta_j|$ across non-zero β_j, where $\mathbf{X^j}$ denotes the j-th column of $\mathbf{X}$.

To simulate the second scenario, we use the same settings as before, but we select the 10 true determinants among those that have a large dot product with W, rather than a small one, and set all α effects to zero.

We apply the three versions of our univariate test described in (1), with $q = 10$ for model (i), to both scenarios over 100 data generations and plot ROC curves (Fig 1 in S1 Appendix). As expected, for the first scenario, the test which does not account for the population structure has very low power to detect patterns associated with the phenotype: by construction, some patterns with zero actual effect have large dot products with $W\alpha$, which inflates the estimate of their effect and leads to false discoveries. Taking the population structure into account in the model improves the power by limiting this inflation. For the second scenario, we observe the opposite effect: correcting for the population structure decreases the power to detect true determinants. Assuming there is a population effect when there is no such effect in reality, leads to artificially deflating the estimated effects of patterns which are associated with the population structure.

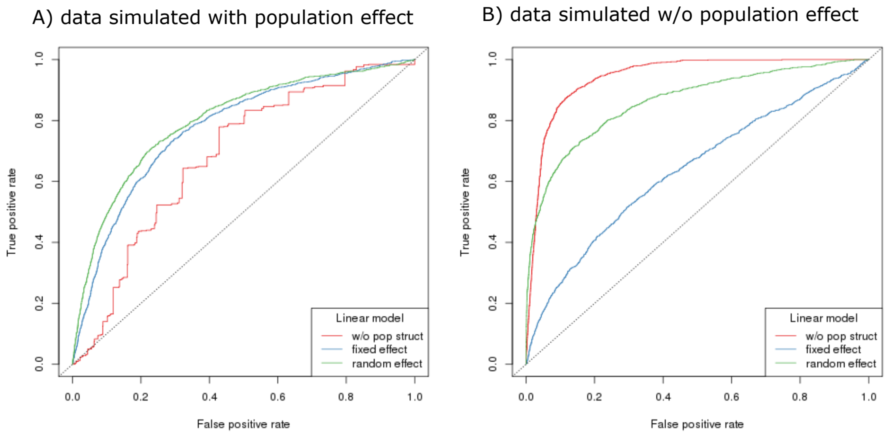

Fig 1. Evaluation of test models on simulated data. Scenarios (A) and (B) intend to illustrate the model ability to detect true positives in the presence or in the absence of a population effect on the observed resistance. In the first case, the ROC curve shows that taking the population structure into account increases the power, while in the second case, correcting for the population structure when there is not, decreases the power to detect true determinants. Using a random effect model is however more robust and leads to a smaller power loss than using a fixed-effect model.

Real data

Using the true phenotype data for both amikacin and levofloxacin resistance in *P. aeruginosa*, we also evaluate a metric based on libraries of known genetic determinants of resistance [6] which we use as our positive set. In this case, we lose the exact knowledge of which unitigs are negative, *i.e.*, have no effect on the phenotype: some selected patterns may not be linked to any know genetic determinant of resistance

just because they are still unreported. Instead of ROC curves, we therefore resort to plotting the true positive rate (TPR) – using identified and hence known positives – as a function of the number of positives called by the method – the false positive rate corresponding to this number being unknown. Assigning each selected pattern to a true or false status requires a mapping step: we choose to identify a pattern as a true determinant if it corresponds to at least one unitig which maps to a known genetic determinant from the resistance gene sequence database [6].

Figs 2A and 3A in S1 Appendix are produced by bugwas, and show the p-value of the test for association of each column of W with the phenotype. In the case of amikacin, two columns are found to have a significant effect at level 0.01, whereas all columns have p-values larger than 0.01 in the case of levofloxacin. Figs 2B and 3B in S1 Appendix show that correcting for population structure increases the proportion of known genetic determinants of resistance to amikacin recovered for every number of predicted positives, but decreases this proportion in the case of levofloxacin.

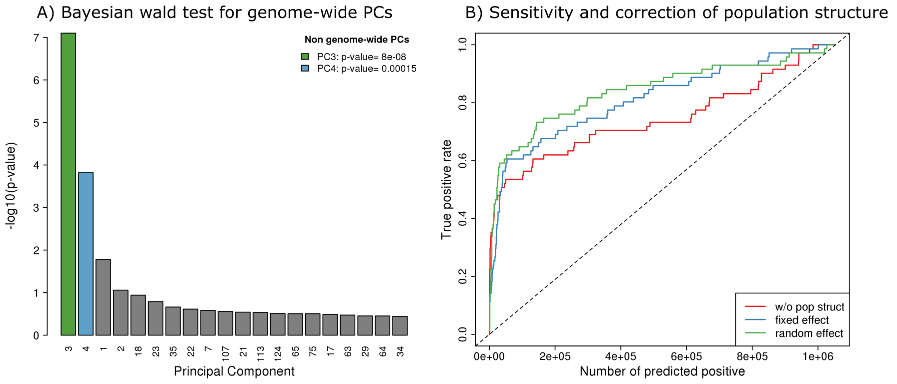

Fig 2. Amikacin resistance and association models. (A) Two PCs were found significantly associated to the *P. aeruginosa* amikacin phenotype. (B) In this case, the random-effect model performs the best. The model which does not account for the population structure effect performs the worst to retrieve genuine variants of amikacin resistance.

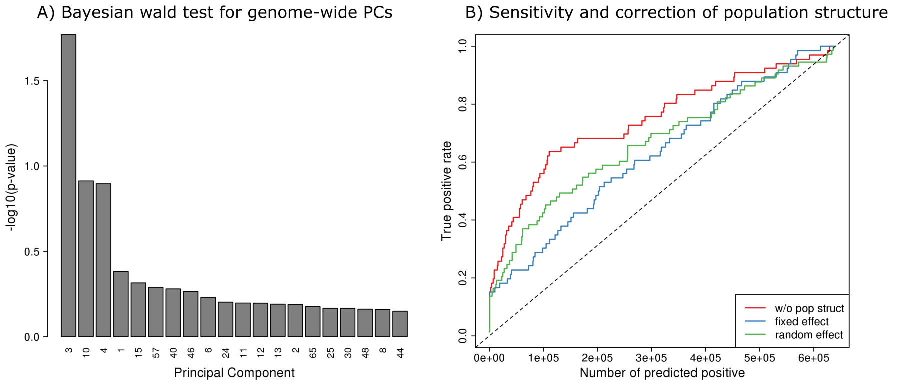

Fig 3. Levofloxacin resistance and association models. (A) No PC was found significantly associated to the P. aeruginosa levofloxacin phenotype. (B) In this case, the model which does not account for the population structure effect performs the best. The linear mixed model still performs better than the fixed effect model to retrieve genuine variants of levofloxacin resistance.

The random effect approach of bugwas is a good choice on both simulated and real

data regardless of the effect of the population structure on the phenotype: it outperforms both the uncorrected and the fixed effect approaches in the presence of a population effect, and is only moderately affected by the absence of such effect. We thus implement this model using the bugwas package in DBGWAS.

References

1. Price AL, Patterson NJ, Plenge RM, Weinblatt ME, Shadick NA, Reich D. Principal components analysis corrects for stratification in genome-wide association studies. Nature genetics. 2006;38(8):904.
2. Chang CC, Chow CC, Tellier LC, Vattikuti S, Purcell SM, Lee JJ. Second-generation PLINK: rising to the challenge of larger and richer datasets. Gigascience. 2015;4(1):7.
3. Yu J, Pressoir G, Briggs WH, Bi IV, Yamasaki M, Doebley JF, et al. A unified mixed-model method for association mapping that accounts for multiple levels of relatedness. Nature genetics. 2006;38(2):203.
4. Kang HM, Zaitlen NA, Wade CM, Kirby A, Heckerman D, Daly MJ, et al. Efficient control of population structure in model organism association mapping. Genetics. 2008;178(3):1709–1723.
5. Earle SG, Wu CH, Charlesworth J, Stoesser N, Gordon NC, Walker TM, et al. Identifying lineage effects when controlling for population structure improves power in bacterial association studies. Nature microbiology. 2016; p. 16041.
6. Jaillard M, van Belkum A, Cady KC, Creely D, Shortridge D, Blanc B, et al. Correlation between phenotypic antibiotic susceptibility and the resistome in *Pseudomonas aeruginosa*. International journal of antimicrobial agents. 2017;.
